# PD-1 Blockade–Induced DKK1 Expression by CD8^+^ T Cells Promotes Blood–Brain Barrier Permeabilization

**DOI:** 10.1158/2159-8290.CD-25-1222

**Published:** 2026-01-13

**Authors:** Abhilash Deo, Sapir Levin, Chen Buxbaum, Madeleine Benguigui, Bar Manobla, Galit Saar, Noam Bosak, Eyal Bergmann, Ayelet Eran, Anat Grinfeld, Michal Harel, Coren Lahav, Ziv Raviv, Sameh Daher, Alona Zer, Julia Helena Reuter, Claus Peter Heuβel, Petros Christopoulos, Keren Yizhak, Yuval Shaked

**Affiliations:** 1 Department of Cell Biology and Cancer Science, Rappaport Faculty of Medicine, Technion - Israel Institute of Technology, Haifa, Israel.; 2 Rappaport Technion Integrated Cancer Center, Technion - Israel Institute of Technology, Haifa, Israel.; 3 Department of Neurology, Rambam Healthcare Campus, Haifa, Israel.; 4 Biomedical Core Facility, Rappaport Faculty of Medicine, Technion-Israel Institute of Technology, Haifa, Israel.; 5 Department of Neuroscience, Monash University, Melbourne, Australia.; 6 Department of Psychiatry, Rambam Healthcare Campus, Haifa, Israel.; 7 Department of Radiology Rambam Healthcare Campus, Haifa, Israel.; 8 Oncohost LTD., Binyamina, Israel.; 9 Department of Oncology, Rambam Healthcare Campus, Haifa, Israel.; 10 Diagnostic and Interventional Radiology with Nuclear Medicine, Thoraxklinik, University of Heidelberg, Heidelberg, Germany.; 11 Translational Lung Research Center (TLRC) at German Center for Lung Research (DZL), Heidelberg, Germany.; 12 Diagnostic and Interventional Radiology, University Hospital Heidelberg, Heidelberg, Germany.; 13 Department of Medical Oncology, Thorax Clinic, Heidelberg, Germany.

## Abstract

Anti–PD-1 therapy benefits a subset of patients with brain metastasis (BrM); however, heterogeneous responses imply an incomplete understanding of the brain−immune ecosystem. To elucidate host-driven determinants of this variability, we performed single-cell RNA sequencing to characterize the brain microenvironment. Although anti–PD-1 induced robust antitumor immune activation, it uniquely, among all immune checkpoint inhibitors (ICI) tested, compromised blood–brain barrier (BBB) integrity. This permeabilization was mediated by DKK1-expressing activated CD8^+^ T cells through the induction of β-catenin/TCF and FOXM1 pathways, contributing to endothelial cell destabilization. Depleting plasma DKK1 restored BBB integrity and reduced experimental BrM formation. Clinically, patients with lung cancer receiving anti–PD-1 exhibited increased magnetic resonance imaging contrast enhancement in the brain, suggestive of BBB perturbations, and increasing plasma DKK1 levels correlated with higher BrM incidence in nonresponders. Sequential administration of anti–PD-1 followed by cisplatin improved intracranial cisplatin delivery and therapeutic efficacy in ICI-resistant BrM. These findings identify anti-PD-1–induced BBB modulation as a tractable vulnerability in BrM management.

**Significance:**

Our study demonstrates that PD-1 blockade induces DKK1 expression in activated CD8^+^ T cells, leading to BBB permeabilization. This previously unrecognized host-driven mechanism may explain heterogeneous intracranial responses to immunotherapy and identifies BBB modulation as a therapeutic opportunity to enhance drug delivery and efficacy for BrM.

*See related commentary by Karreman and Winkler, p. 831*

## Teaser

CD8^+^ T-cell–derived DKK1 mediates anti−PD1-induced opening of the blood–brain barrier, with clinical implications for brain metastasis risk and therapeutic exploitation in the resistant setting.

## Introduction

Brain metastasis (BrM) is a prevalent clinical complication, affecting 9% to 40% of patients with cancer, and is associated with a dismal 5-year survival rate of 2.4% across all types of primary tumors (1). Although traditional systemic therapies have demonstrated limited efficacy in targeting intracranial tumors, immune checkpoint inhibitors (ICI) have emerged as a promising therapeutic approach, showing significant benefits in BrM, particularly among patients with non–small cell lung carcinoma (NSCLC) and melanoma (2–4). Their ability to reduce intracranial tumor burden and improve response rates has made ICIs an increasingly favored adjuvant treatment, complementing other modalities like radiation and surgery (5). Additionally, the clinical use of ICIs is expanding to other malignancies, including head and neck, non–clear-cell renal, and triple-negative breast cancers (6–8).

Although most clinical studies highlight the intracranial efficacy of ICIs, therapeutic responses in BrM remain highly heterogeneous (2, 9). These variable outcomes present not only a clinical challenge but also an opportunity to better understand and optimize ICI-based therapies. Among the various factors affecting ICI responses in the brain, a particularly critical determinant is the unique immune dynamics of the central nervous system (CNS; ref. 10). Although historically considered an immune-privileged site, the CNS is now recognized as capable of mounting effective immune responses. ICIs can cross the blood–brain barrier (BBB) to a limited extent though their antitumor effects are primarily mediated by activated peripheral T cells that infiltrate the brain parenchyma and cerebrospinal fluid (CSF; refs. 11, 12). Moreover, sustained PD-1 receptor occupancy on circulating T cells by nivolumab has been linked to intracranial responses (13). The intracranial efficacy of ICIs is therefore believed to result less from direct tumor penetration and more from systemic immune activation that remodels the CNS immune microenvironment. Preclinical studies support this mechanism, showing that ICIs enhance systemic immune activation, expand homing-competent CD8^+^ effector T cells, and upregulate endothelial adhesion molecules such as ICAM-1 and VCAM-1 on tumor vasculature, facilitating T-cell entry into the brain (14). These findings highlight the therapeutic potential of ICIs for BrM while indicating the critical need for a more comprehensive understanding of the brain–immune interface in the context of heterogeneous responses.

Our previous study demonstrated that ICIs can elicit host-driven systemic effects that extend beyond direct cancer cell killing, increasing metastatic behavior and resistance to therapy (15). However, these host-derived protumorigenic effects have been characterized primarily in peripheral tumors, whereas the impact of PD-1 blockade on the CNS and the development of BrM remains unexplored. Given that intracranial benefit from ICIs depends on peripheral T-cell activation, trafficking, and effective entry into the CNS, dissecting how anti–PD-1 therapy shapes the brain–immune interface under the constraints of the BBB is essential for maximizing therapeutic efficacy against intracranial tumors.

Here, we employed a comprehensive approach to investigate host-dependent mechanisms by which ICIs function within the brain. We found that anti–PD-1 treatment enhances antitumor immunity in the brain while also impairing BBB integrity. This permeabilization was mediated by DKK1 secreted from activated CD8^+^ T cells, potentially facilitating cellular transmigration into the brain. Extending the clinical relevance of these findings, magnetic resonance imaging (MRI) data revealed increased BBB permeability in patients with BrM-free lung cancer receiving anti–PD-1 therapy. Additionally, we demonstrated that implementing a combinatorial treatment schedule of anti–PD-1 therapy followed by chemotherapy resulted in enhanced intracranial efficacy of chemotherapy, translating into improved survival outcomes.

## Results

### Defining the Immune Cell Landscape Underlying the Heterogeneous Response of BrM to Anti–PD-1 Therapy

**Figure 1.**
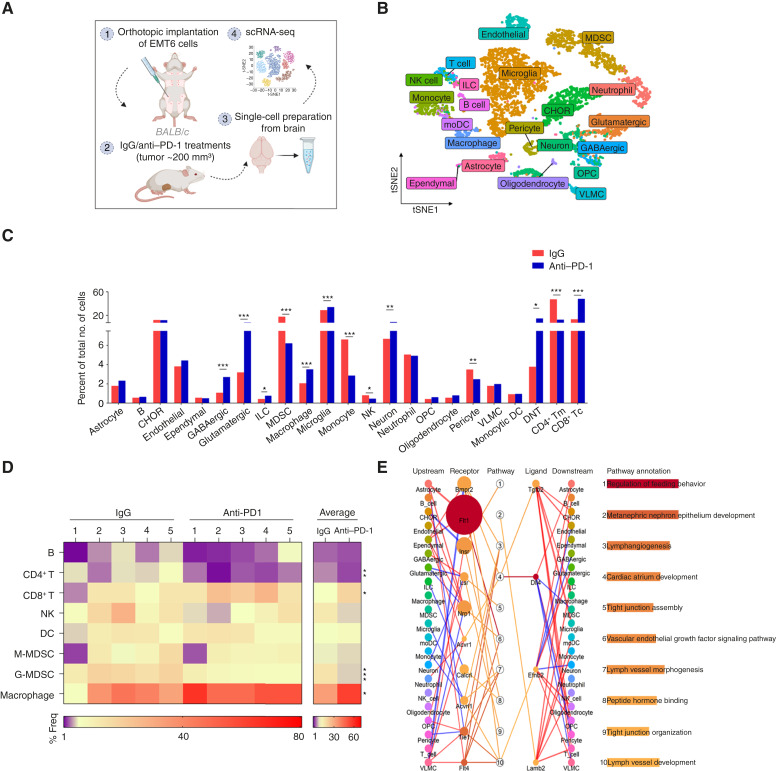
PD-1 blockade reprograms the brain–immune–vascular microenvironment. **A–C,** Eight-week-old BALB/c mice harboring orthotopic EMT6 tumors were treated with anti–PD-1 or IgG control. After 1 week, mice were perfused; brains were harvested and prepared as a single-cell suspension for scRNA-seq. A schematic of the experimental design is shown (**A**). A t-distributed stochastic neighbor embedding (tSNE) plot of 21 cell clusters identified in the scRNA-seq analysis of the brain. Cells from different clusters are marked by colors (**B**). A bar plot shows the relative abundance of immune, neuronal, and nonneuronal populations in the brains of IgG- and anti-PD-1–treated mice, quantified as percentages of total cells (*n* = 5 mice pooled/group; **C**). **D,** Heatmaps displaying the individual and average percentages of various immune cell populations using flow cytometry in the brains of IgG- and anti-PD-1–treated mice. **E,** A pathway-mediated cell–cell communication network of endothelial cells in the scRNA-seq data. Shown are the top 10 significantly upregulated pathways in endothelial cells in IgG compared with anti-PD-1–treated groups. The line widths connecting the upstream/downstream and receptor/ligand columns depict the overall interaction intensity between various cell types and endothelial cells through specific receptors or ligands. The colors signify whether the interaction intensity is upregulated (red) or downregulated (blue) in IgG compared with anti-PD-1–treated groups; the sizes and colors of dots in the receptor/ligand column represent the average log_2_FC and −log_10_(*P*) of receptors’ expression in endothelial cells compared with all other cells in the IgG-treated group. Significance was assessed by means of the bias-corrected and accelerated bootstrap method for **C** and the Student *t* test for **D** (*, *P* < 0.05; **, *P* < 0.01; ***, *P* < 0.001). CHOR, choroid plexus epithelial cells; ILC, innate lymphoid cells; MDSC, myeloid derived suppressor cells; G-MDSC, granulocytic-MDSC; M-MDSC, monocytic-MDSC; NK, natural killer cells; OPC, oligodendrocyte precursor cells; VLMC, vascular leptomeningeal cells; DC, dendritic cells; DNT, double-negative T; Tc, T cytotoxic; Tm, T memory; BCa, bias-corrected and accelerated. (**A,** created in BioRender. Raviv, Z. (2026) https://BioRender.com/znweqpc.)

The progressive behavior of BrM may be simultaneously governed by the unrestricted proliferation of cancer cells that have escaped therapy and tumor-conducive host effects induced in response to ICI, as previously reported (15). To further dissect how the host-triggered response to anti–PD-1 modulates the brain–immune ecosystem, we performed single-cell RNA sequencing (scRNA-seq) on tumor-naive brains of anti-PD-1–treated mice bearing EMT6 breast carcinoma (Fig. 1A, for illustration), which partially and spontaneously responds to anti–PD-1 and reflects clinical heterogeneity (16). Supervised clustering of scRNA-seq data identified 21 cell populations, including endothelial cells, immune cells, and neuronal and nonneuronal subtypes of brain cells (Fig. 1B; Supplementary Fig. S1; Supplementary Table S1). The absence of luciferase transcripts in scRNA-seq data confirmed a metastasis-free brain microenvironment. Compared with IgG-treated controls, anti-PD-1–treated brains harbored increased CD8^+^ T cells and macrophages, reduced CD4^+^ T cells, NK cells, granulocytic myeloid-derived suppressor cells (G-MDSC), monocytic MDSCs (M-MDSC), and unchanged B cells and dendritic cells (DC; Fig. 1C). Flow cytometry analysis largely corroborated these trends, except for NK cells, in which differences were not statistically significant (Fig. 1D; Supplementary Fig. S2 for flow cytometry gating strategy and Supplementary Fig. S3A–S3H). These data suggest that anti–PD-1 primes the premetastatic brain, enhancing antitumor immune cell infiltration in the absence of overt disease.

Surprisingly, our scRNA-seq data analysis revealed two distinct MDSC subpopulations between IgG- and anti-PD-1–treated brains (Supplementary Fig. S4A), as assessed by differential abundance, suggesting a treatment-induced shift in myeloid cell states. MDSCs from anti-PD-1–treated brains exhibited upregulation of lipocalin-2 (LCN2; Supplementary Fig. S4B), a prometastatic protein known to activate astrocytes and recruit additional myeloid cells, thereby promoting BrM development (17). Elevated LCN2 was also detected in plasma and GR1^+^ cell–conditioned media from anti-PD-1–treated mice (Supplementary Fig. S4C), as well as in brain-infiltrating granulocytes, compared with their IgG-treated counterparts (Supplementary Fig. S4D). Overall, systemic anti–PD-1 treatment drives effector T-cell recruitment into the brains of mice without established BrM, demonstrating immunologic responsiveness to peripheral checkpoint blockade. However, the concomitant accumulation and transcriptional reprogramming of MDSCs suggest an early onset of counteractive host responses that may blunt anti–PD-1 efficacy.

### Compromised BBB Function Following Anti–PD-1 Treatment in Mice

ICIs, although effective in some patients, can elicit complex and sometimes unexpected responses within the brain. To investigate cellular changes that may underlie heterogeneous responses, we analyzed alterations in the brain microenvironment following anti–PD-1 therapy. Unexpectedly, we observed a striking 1.8-fold increase in innate lymphoid cell (ILC) infiltration and a marked loss of pericytes in anti-PD-1–treated mice compared with IgG-treated controls (Fig. 1C). ILCs, typically confined to the meninges and choroid plexus, are restricted from entering the parenchyma by the blood–CSF and meningeal barriers, along with the BBB (18). Their infiltration, along with pericyte loss, is known to impair BBB function (19, 20) and suggests that anti–PD-1 may inadvertently compromise barrier function.

Given the central role of endothelial cells in BBB maintenance (20), we conducted a communication chain analysis focused on endothelial interactions within the scRNA-seq dataset (Fig. 1E). Unlike endothelial cells in anti-PD-1–treated brains, IgG-treated brains displayed a significant upregulation of pathways associated with the organization and assembly of tight junctions (top 10; Fig. 1E). Additionally, endothelial cells from anti-PD-1–treated brains exhibited increased interactions with neurons, particularly glutamatergic neurons, via neuregulin-1 and lipolysis-stimulated lipoprotein receptors, a tight junction component downregulated in leaky CNS vessels (Fig. 1E; Supplementary Table S2; ref. 21). We also observed enhanced cross-talk between endothelial cells and microglia/monocytes through TGF-β1–ACVRL1 (ALK1) signaling, a pathway implicated in BBB repair (22), and with GABAergic neurons via ephrin-B2, known to mediate neurovascular remodeling (Fig. 1E; Supplementary Table S2; ref. 23). Taken together, these data suggest potential BBB remodeling.

**Figure 2.**
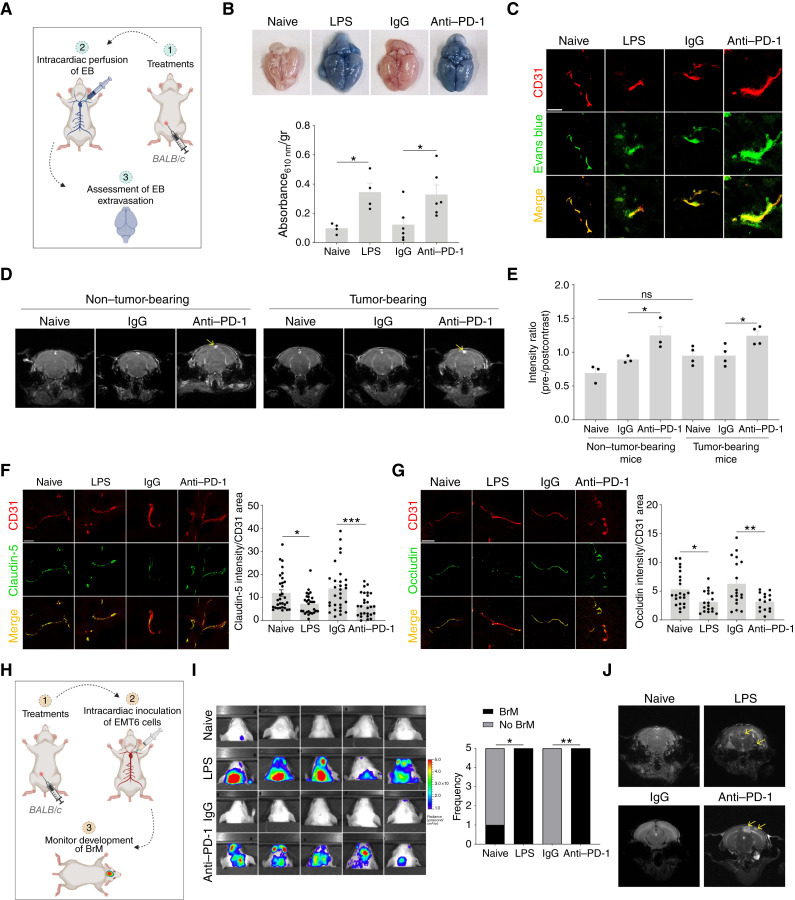
Barrier function of the brain is perturbed following anti–PD-1 therapy. **A,** Scheme illustrating the EB assay performed to evaluate BBB permeability. **B,** Images of EB-perfused brains harvested from naive (untreated), LPS-, IgG-, and anti-PD-1–treated 8-week-old BALB/c mice (*n* = 4–6 mice/group). Absorbance of EB dye extracted per gram of brain tissue is plotted. **C,** Immunofluorescence images showing colocalization of CD31 (blood vessels, red) and EB (green) in the brains of naive (untreated), LPS-, IgG-, and anti-PD-1–treated BALB/c mice. Scale bars, 20 µm. **D** and **E,** Eight-week-old BALB/c mice, either non–tumor-bearing or tumor-bearing orthotopic EMT6 breast tumors, were left untreated or treated with IgG or anti–PD-1. Representative DCE-MRI images of mice from different treatment groups are shown (*n* = 3–4 mice/group; **D**). Change in gadolinium intensity at 30 minutes after injection is plotted as the ratio of pre-contrast (*t* = 0, prior to gadolinium injection) to post-contrast signal intensity (**E**). Arrows indicate regions of increased gadolinium enhancement in the venous sinus area of the anti-PD-1–treated mice. **F** and **G,** Immunofluorescence images showing colocalization of CD31 (blood vessels, red) with the tight junction proteins claudin-5 (**F**, green) and occludin (**G**, green) in the different treatment groups. The distribution of tight junction proteins along blood vessel surfaces is plotted. Scale bars, 20 µm. **H,** Scheme illustrating the BrM assay used to assess BrM development as a function of BBB permeability. **I,** Eight-week-old BALB/c mice were pre-treated with LPS, IgG, anti–PD-1, or left untreated (naive), followed by intracardiac injection of luciferase-tagged EMT6 cells. Mice were then monitored for the development of BrM using bioluminescence imaging. Shown are bioluminescence images of all mice (*n* = 5 mice/group). The frequency of metastasis in each group is quantified and plotted. **J,** Representative MRI scans showing brain tumors: In LPS-treated mice, lesions were detected in the corpus callosum and left temporal lobe; in anti-PD-1–treated mice, tumors were localized to the cortical region and the left parasagittal area of the parietal lobe. Arrows indicate the tumor lesions. Significance was assessed using one-way ANOVA for **B** and **E**–**G** and Fisher’s exact test for **I** (*, *P* < 0.05; **, *P* < 0.01; ***, *P* < 0.001). [**A,** Created in BioRender. Raviv, Z. (2026) https://BioRender.com/urq64oq; **H,** Created in BioRender. Raviv, Z. (2026) https://BioRender.com/mqjamit.]

Computational analysis suggested that anti–PD-1 disrupts BBB stability through host-driven cellular interactions. To validate this, non–tumor-bearing mice were preconditioned with anti–PD-1 or IgG, and BBB permeability was assessed. The use of non−tumor-bearing mice was carried out to delineate the direct host response to PD-1 blockade, independent of tumor–immune interactions, thereby solely isolating the systemic and CNS-specific effects of therapy, as previously demonstrated (15). BBB permeability, assessed by the Evans Blue (EB) assay (Fig. 2A, for illustration), was markedly increased in anti-PD-1–treated mice, comparable with lipopolysaccharide (LPS) controls used as positive controls (Fig. 2A and B). Immunostaining of blood vessels marked by CD31^+^ on these EB-perfused brain sections displayed an overt extravasation of EB dye into the surrounding tissue in anti-PD-1–treated and LPS-treated groups, contrary to untreated or IgG-treated groups, wherein the EB dye was confined well within the vessel’s lumen (Fig. 2C). Consistently, longitudinal dynamic contrast enhancement MRI (DCE-MRI) showed significantly greater gadolinium enhancement in anti-PD-1–treated mice versus controls, particularly in regions adjacent to the sinuous veins (Fig. 2D and E; Supplementary Fig. S5A), indicating increased BBB permeability. Notably, similar BBB permeabilization was observed in mice bearing EMT6 breast tumor xenografts (with no BrM), supporting a systemic host response rather than a tumor-dependent effect. Of note, the baseline gadolinium enhancement in tumor-bearing mice was slightly higher than that found in non–tumor-bearing mice as also assessed using EB assay; however, these results were not significant (Fig. 2D and E ; Supplementary Fig. S5A and S5B).

It should be noted that similar effects on BBB opening were observed using a different anti–PD-1 clone (29F.1A12; Supplementary Fig. S6A) and with alternative delivery routes of EB administration (tail vein vs. intracardiac; Supplementary Fig. S6B), ruling out clone- or route-specific effects. Similarly, the murine anti–PD-1 antibody also induced BBB permeability (Supplementary Fig. S6C), indicating that the effect was not mediated by antibody-dependent cellular cytotoxicity. As anti–PD-L1, anti–PD-L2, or anti-CTLA4 before treatment did not affect BBB permeability (Supplementary Fig. S6A), PD-1 blockade was identified as the sole driver of this phenotype.

Furthermore, analysis of tight junction proteins, for example, claudin-5 and occludin in endothelial cells, which have been shown to act as structural barriers in the BBB (24), revealed lower structural overlap with blood vessels in LPS and anti-PD-1–treated groups than in untreated or control groups (Fig. 2F and G). Taken together, these results explicitly indicate compromised barrier function after anti–PD-1 treatment.

To evaluate whether BBB disruption promotes circulating cancer cell entry into the brain, non–tumor-bearing mice pretreated with anti–PD-1 or IgG were subsequently injected intracardially with luciferase-tagged EMT6 or Lewis lung carcinoma (LLC) cells to assess the functional integrity of the BBB. The development of BrM was monitored by bioluminescence imaging, and metastasis frequency was assessed at the earliest detectable intracranial signal (∼2 weeks after inoculation; Fig. 2H, for illustration). Anti–PD-1 preconditioning increased BrM frequency, similar to LPS-treated controls (Fig. 2I; Supplementary Fig. S7). Moreover, MRI confirmed intracranial tumor growth (EMT6 cells), with lesions in anti-PD-1–treated mice localized to the cortical and left parasagittal regions of the parietal lobe versus the corpus callosum and temporal lobe in LPS-treated animals (Fig. 2J). These results indicate that BBB opening following anti–PD-1 treatment facilitates greater entry of circulating cancer cells, one of the key events in the metastatic cascade leading to BrM.

### DKK1 Mediates BBB Permeabilization Following Anti–PD-1 Therapy

**Figure 3.**
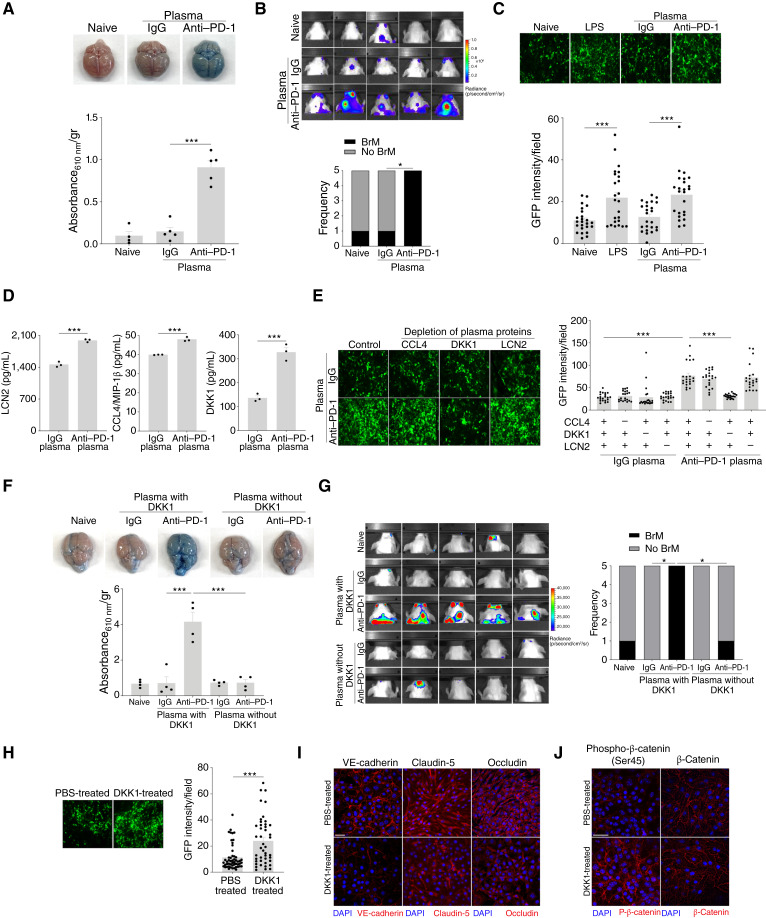
Circulating DKK1 accounts for anti-PD-1–induced BBB opening. Plasma was obtained from naive, IgG-, and anti-PD-1–treated mice and used for a variety of assays as described below. **A,** Representative images of EB-perfused brains of 8-week-old BALB/c mice subjected to plasma treatments (*n* = 4–5 mice/group). Absorbance of EB dye extracted per gram of brain tissue is plotted. **B,** Bioluminescence images of the brains of 8-week-old BALB/c mice subjected to different plasma treatments in the experimental BrM assay (*n* = 5 mice/group). The frequency of metastasis is plotted. **C,** Representative images showing the migrated fraction of EMT6 cells tagged with GFP, assessed through the trans-endothelial cell migration assay under different plasma conditions, as indicated in the figure. Migrated fractions of cancer cells are plotted. **D,** Eight-week-old BALB/c mice were treated with IgG or anti–PD-1 for 1 week, after which plasma was collected. The upregulation of potential BBB-disrupting factors, including LNC2, CCL4/MIP-1β, and DKK1, identified through a cytokine array in anti–PD-1 plasma compared with IgG, was validated using specific ELISAs (*n* = 3 mice/group). A bar graph showing quantification is plotted. **E,** Representative images showing the migrated fraction of EMT6 cells in a trans-endothelial cell migration assay under IgG and anti–PD-1 plasma–treated conditions, with and without depletion of CCL4, DKK1, or LNC2. The migrated fractions of cancer cells are plotted. **F,** Representative images of EB-perfused brains from BALB/c mice subjected to plasma treatment obtained from naive (untreated), IgG plasma, and anti–PD-1 plasma treatments, both with and without DKK1 depletion (*n* = 4 mice/group). The absorbance of EB dye extracted per gram of brain tissue is plotted. **G,** Bioluminescence images of the brains of BALB/c mice, depicting various treatments as mentioned above, showing BrM (*n* = 5 mice/group). The frequency of metastasis is plotted. **H,** Representative images of EMT6 cells, 48 hours after they underwent trans-endothelial migration under control (PBS) and DKK1-treated (100 ng/ml). The corresponding quantification of migrated cells is plotted. **I** and **J,** Representative immunofluorescence images of bEnd.5 brain endothelial cells showing changes in adherens junction (VE-cadherin, red) and tight junction (claudin-5 and occludin, red) protein expression levels (**I**), together with total and phosphorylated β-catenin (Ser45, red), indicative of WNT pathway suppression following 24 hours of treatment with recombinant DKK1 (100 ng/mL) or PBS (control). Scale bars, 30 µm. Significance was assessed using one-way ANOVA for **A**, **C**, **E**, and **F**; Fisher’s exact test for **B** and **G**; and Student *t* test for **D** and **H** (*, *P* < 0.05; ***, *P* < 0.001).

To further elucidate the mechanism(s) underlying BBB permeabilization, we tested whether systemic factors elicited by anti–PD-1 therapy drive BBB perturbations. BBB integrity was evaluated *in vivo* using plasma from anti-PD-1–treated or IgG-treated mice. To exclude direct antibody effects, circulating immunoglobulins were depleted using sepharose beads. Remarkably, untreated mice transfused with anti–PD-1 plasma developed BBB leakage, as evidenced by EB extravasation, and showed infiltration of cancer cells in the brain, unlike those receiving IgG plasma or untreated controls (Fig. 3A and B). These findings implicate plasma-borne, treatment-induced factors in compromising the BBB. *In vitro*, we employed antibody-depleted plasma in a trans-endothelial migration assay, mimicking the BBB. GFP-labeled EMT6 (EMT6-GFP) cells and brain endothelial monolayers were independently pretreated with antibody-depleted plasma to replicate *in vivo* exposure. Transmigration across untreated endothelium defined baseline migration, whereas LPS-treated endothelium served as a positive control. Under anti–PD-1 plasma conditions, EMT6-GFP cells exhibited significantly enhanced transmigration compared with both IgG plasma and untreated conditions (Fig. 3C), indicating functional endothelial barrier disruption. Although we did not directly assess the impact of plasma on endothelial cell death or migration, plasma treatments to individual cell types revealed that enhanced cancer cell transmigration resulted from endothelial barrier loss rather than increased invasive potential of cancer cells exposed to anti–PD-1 plasma factors (Supplementary Fig. S8A).

Given that changes in circulating plasma proteins following anti–PD-1 therapy may affect BBB function, we compared the plasma protein profiles in anti-PD-1–treated and IgG-treated mice to identify candidates that could contribute to this effect. Indeed, anti–PD-1 plasma exhibited elevated levels of several known BBB-disrupting proteins, including DKK1, LCN2, and CCL4 (25–27), compared with IgG plasma (Supplementary Fig. S8B and Supplementary Table S3 for the cytokine array results and Fig. 3D for validation). The depletion of DKK1 in anti–PD-1 plasma rescued the barrier function of endothelial cells *in vitro*, whereas the individual depletion of LCN2 and CCL4 in anti–PD-1 plasma had no effect (Fig. 3E). Furthermore, DKK1 depletion in anti–PD-1 plasma affected only the endothelial cells and not cancer cells, consistent with a barrier-specific mechanism (Supplementary Fig. S8C). Corroborating the *in vitro* results, mice receiving DKK1-depleted anti–PD-1 plasma showed improved BBB function, evidenced by reduced EB extravasation (Fig. 3F) and decreased BrM frequency compared with matched controls (Fig. 3G). These findings identify DKK1 as a key host-derived regulator of BBB permeability in the context of anti–PD-1 therapy.

To further support our findings, recombinant DKK1 enhanced cancer cell transmigration *in vitro* (Fig. 3H), indicating disruption of the endothelial barrier. Consistently, DKK1 treatment reduced the endothelial expression of the junctional proteins VE-cadherin, claudin-5, and occludin (Fig. 3I; Supplementary Fig. S9A–S9C). As DKK1 antagonizes WNT signaling, a pathway critical for maintaining tight and adherens junction integrity in endothelial cells, we evaluated β-catenin, a key downstream effector of this pathway, in which loss in endothelial cells is known to increase transcellular permeability (28). DKK1 treatment markedly increased Ser45-phosphorylated β-catenin (targeted for degradation) with no change in total β-catenin levels (Fig. 3J; Supplementary Fig. S9D and S9E), indicating WNT pathway suppression. Mechanistically, these findings reveal that DKK1 disrupts endothelial barrier function by impairing the WNT–β-catenin–junctional protein axis.

### Cytotoxic T Cells Actively Contribute to Anti-PD-1–Mediated BBB Permeabilization

**Figure 4.**
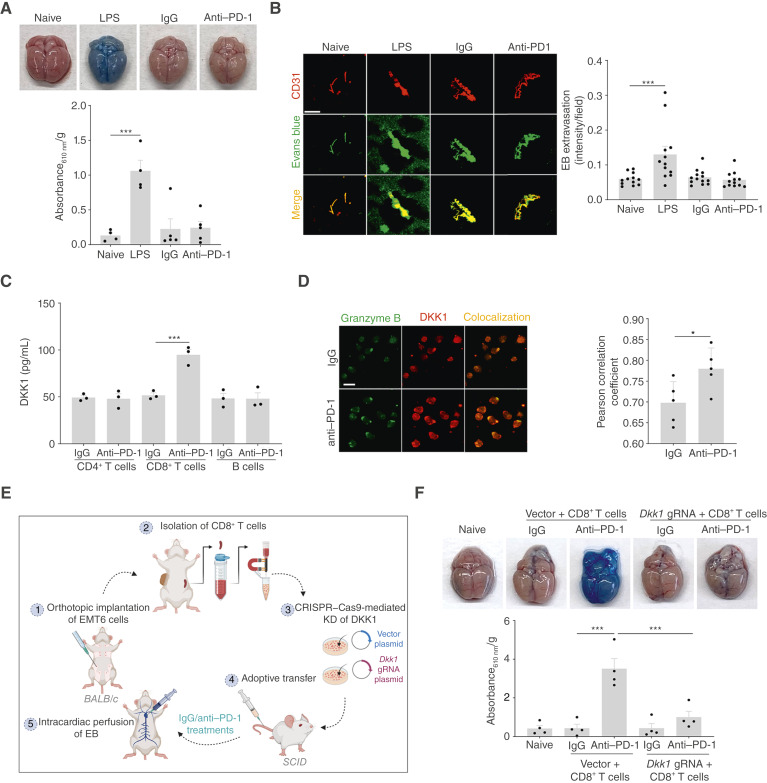
Depletion of CD8^+^ T cells expressing DKK1 restores BBB function and mitigates BrM. Eight-week-old SCID mice were treated with LPS, IgG, or anti–PD-1. After 1 week, mice were perfused with EB, and subsequently, brains were harvested. **A,** Representative images of EB-perfused brains (*n* = 4–5 mice/group). Absorbance of EB dye extracted per gram of brain tissue is plotted. **B,** Representative immunofluorescence images showing colocalization of CD31 (blood vessels, red) and EB (green) in the brains from the different groups. Extravasation of EB per field is plotted. Scale bars, 20 µm. **C,** DKK1 levels were analyzed using a specific ELISA in the conditioned medium of CD4^+^ T, CD8^+^ T, and B cells harvested from the spleens of IgG and anti-PD-1–treated EMT6 tumor–bearing BALB/c mice. **D,** Representative immunofluorescence images showing colocalization of granzyme B (green) and DKK1 (red) in CD8^+^ T cells harvested from IgG and anti-PD-1–treated BALB/c mice. The colocalization coefficient is plotted. Scale bars, 10 µm. **E** and **F,** Adoptive transfer of wild-type (Vector) and *Dkk1*-KD [guide RNA (gRNA)] CD8^+^ T cells obtained from BALB/c mice was performed on 8-week-old SCID mice, followed by IgG or anti–PD-1 treatment. At the endpoint, the mice were perfused with EB dye. A scheme of the experimental design is illustrated (**E**). Representative images of EB-perfused brains taken from mice under the same treatment groups (*n* = 4 mice/group) are shown, and the absorbance of EB dye extracted per gram of brain tissue is plotted (**F**). Significance was assessed using one-way ANOVA for **A**–**C** and **F** and Student *t* test for **D** (*, *P* < 0.05; ***, *P* < 0.001). [**E,** Created in BioRender. Raviv, Z. (2026) https://BioRender.com/rwrqpz8.]

We next sought to identify the cellular source of circulating DKK1 responsible for BBB disruption. As the anti–PD-1 antibody primarily targets T cells (29), we tested whether lymphocytes were required for BBB perturbation. In severe combined immunodeficient (SCID) mice, deficient in B and T lymphocytes (29), anti–PD-1 treatment failed to induce BBB permeability (Fig. 4A and B), in contrast to immunocompetent mice (Fig. 2B and C). Notably, LPS still induced BBB disruption in SCID mice, indicating a distinct mechanism from that of anti–PD-1. The potential involvement of the adaptive immune system was further supported by a transfusion experiment, in which plasma from anti-PD-1–treated BALB/c mice induced BBB permeability in naive SCID recipients (Supplementary Fig. S10).

Next, to pinpoint the relevant immune subset, we isolated CD4^+^ T cells, CD8^+^ T cells, and B cells from the spleens of EMT6 tumor−bearing mice treated with IgG or anti–PD-1 and measured DKK1 levels in their conditioned media using ELISA. Although all cell types secreted basal levels of DKK1, CD8^+^ T cells displayed the most pronounced increase upon anti–PD-1 treatment (Fig. 4C). Immunostaining further confirmed elevated DKK1 within granzyme B^+^ secretory granules of CD8^+^ T cells exposed to anti–PD-1, as indicated by the higher colocalization coefficient (Fig. 4D). To functionally validate these findings, CD8^+^ T cells from EMT6 tumor–bearing mice subjected to CRISPR−Cas9-mediated *Dkk1*-knockdown (KD; Supplementary Fig. S11A for the CRISPR−Cas9 map and Supplementary Fig. S11B for KD validation) were adoptively transferred into SCID mice, followed by 1 week of anti–PD-1 or IgG treatment (Fig. 4E for illustration). The EB assay in these mice revealed that *Dkk1*-deficient CD8^+^ T cells fail to induce BBB permeability upon anti–PD-1 exposure, unlike wild-type controls (Fig. 4F). Collectively, these results establish CD8^+^ T cells as the primary source of DKK1 during anti–PD-1 therapy and identify secretory granules as the mechanism of delivery for this BBB-disrupting factor.

### PD-1 Blockade Drives DKK1 Expression in CD8^+^ T Cells via β-Catenin/T-cell Factor and FOXM1 Signaling

BBB permeabilization occurred specifically with PD-1 blockade and not with PD-L1 or PD-L2 blockade (Supplementary Fig. S6), suggesting that the degree of T-cell activation, primarily governed by PD-1 signaling, may regulate DKK1 induction. To test this hypothesis, we assessed granzyme B expression and DKK1 secretion in chemically activated CD8^+^ T cells cocultured with LLC cells. Phorbol 12-myristate 13-acetate and ionomycin were used to robustly activate T cells and induce downstream signaling independently of the T-cell receptor (30). LLC cells express high levels of PD-L1 and moderate levels of PD-L2 (Supplementary Fig. S12A) and can block PD-1 signaling on T cells; therefore, they can serve as an appropriate model to evaluate PD-1 pathway–dependent regulation of DKK1. We used two anti–PD-1 clones (RMP1-14 and 29F.1A12) to exclude clone-specific effects and anti-CTLA4 as a negative control, as it neither activates T cells via PD-L1/L2 blockade nor induces BBB permeability *in vivo* (Supplementary Fig. S6).

**Figure 5.**
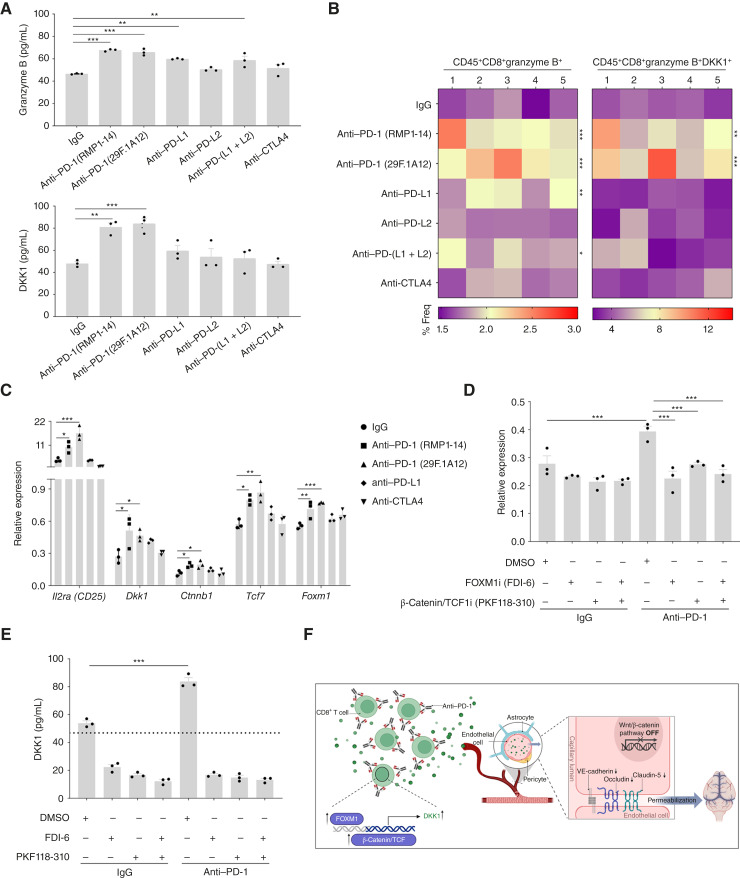
β-Catenin/TCF and FOXM1 signaling regulate *Dkk1* expression in CD8^+^ T cells. CD8^+^ T cells were isolated from the spleens of 8-week-old C57BL/6 mice, activated with phorbol 12-myristate 13-acetate (PMA; 10 ng/mL) and ionomycin (10 µg/mL), and cocultured with LLC tumor cells under various ICI-treated conditions, as indicated in the figure. **A,** Secretory levels of granzyme B and DKK1 in the conditioned media of CD8^+^ T cells were measured using specific ELISA. **B,** Flow cytometry was performed to assess activated CD8^+^ T cells (CD45^+^CD8^+^granzyme B^+^) and DKK1^+^ activated CD8^+^ T cells (CD45^+^CD8^+^granzyme B^+^DKK1^+^). **C,** CD8^+^ T cells were isolated and activated with PMA and ionomycin and cultured under various ICI-treated conditions, as indicated in the figure. Cells were assessed for RNA expression of various factors, as indicated in the figure, using real-time PCR. **D** and **E,** CD8^+^ T cells were isolated and activated with PMA and ionomycin, followed by treatments with a FOXM1 inhibitor (FDI-6; 30 µM), a β-catenin/TCF inhibitor (PKF118-310; 1 µM), a combination of both, or a vehicle control (DMSO) for 24 hours. After treatment, cells were harvested to analyze (**D**) *Dkk1* expression by real-time PCR and (**E**) DKK1 secretion levels (from conditioned medium) using a specific ELISA (the dotted line represents the detection limit). **F,** Illustration summarizing that anti–PD-1 blockade activates CD8^+^ T cells, leading to increased *Dkk1* expression and secretion via β-catenin/TCF and FOXM1 signaling pathways. Secreted DKK1 impairs endothelial barrier integrity by suppressing WNT/β-catenin signaling, resulting in the downregulation of adherens junction (VE-cadherin) and tight junction proteins (claudin-5 and occludin), thereby increasing BBB permeability. Significance was assessed using one-way ANOVA (*, *P* < 0.05; **, *P* < 0.01; ***, *P* < 0.001). [**F,** Created in BioRender. Raviv, Z. (2026) https://BioRender.com/rod21ab.]

Both anti–PD-1 and anti–PD-L1 significantly increased granzyme B secretion, with PD-1 blockade inducing stronger activation (Fig. 5A). Although combined PD-(L1 + L2) blockade caused similar activation to PD-L1 alone, anti–PD-L2 and anti-CTLA4 had minimal effects relative to the IgG-treated control. Flow cytometry analysis further confirmed a greater proportion of activated CD8^+^ T cells (CD45^+^CD8^+^granzyme B^+^) under anti–PD-1 treatment relative to PD-L1 blockade, across both clones tested (Fig. 5B; Supplementary Fig. S12B for gating strategy and Supplementary Fig. S13A). These results indicate that PD-1 blockade induces maximal T-cell activation, whereas PD-L1 blockade shows a weaker effect, a finding consistent with prior reports showing superior clinical outcomes with anti–PD-1 over anti–PD-L1 therapy (31). Our results show that DKK1 secretion was pronounced after PD-1 blockade but marginal under PD-L1 inhibition (Fig. 5A), mirroring the frequency of DKK1^+^ activated CD8^+^ T cells (CD45^+^CD8^+^granzyme B^+^DKK1^+^; Fig. 5B; Supplementary Fig. S13B) and suggesting activation-dependent DKK1 upregulation. Consistent with protein data, PD-1 blockade induced robust *Dkk1* transcription, whereas PD-L1 and CTLA4 blockade did not (Fig. 5C). CD25 (*Il2ra*) expression paralleled these trends (Fig. 5C), reinforcing the link between activation status and *Dkk1* expression and also indicating that anti–PD-1 treatment causes transcriptional activation of *Dkk1*. Of note, the lack of significant activation with anti–PD-L1 (Fig. 5C) may be due to low PD-L1 expression in the *in vitro* system.

*Dkk1* is a well-established target of the β-catenin/T-cell factor (TCF) signaling pathway (32) and is also regulated by the transcription factor FOXM1, the latter being elevated in activated and mature T cells (33). PD-1 blockade upregulated both the β-catenin/TCF and FOXM1 signaling pathways, as evidenced by increased transcript levels of *Ctnnb1*, *Tcf7*, and *Foxm1* (Fig. 5C). Pharmacologic inhibition of β-catenin/TCF and FOXM1, either individually or in combination, not only downregulated *Dkk1* transcription but also resulted in complete abrogation, that is, below the detection level of DKK1 secretion in anti-PD-1–treated CD8^+^ T cells (Fig. 5D and E). Consistently, CRISPR−Cas9-mediated *Ctnnb1*-KD (Fig. S14A for validation) abolished *Dkk1* expression (Supplementary Fig. S14B), establishing β-catenin–mediated transcriptional control. Collectively, these findings demonstrate that PD-1 blockade specifically drives transcriptional activation of *Dkk1* in CD8^+^ T cells via the β-catenin/TCF and FOXM1 pathways. This mechanistic axis explains how immune checkpoint inhibition reshapes T-cell function to modulate BBB integrity (Fig. 5F).

### MRI Reveals Signs of BBB Permeabilization in Patients with Lung Cancer Treated with Anti–PD-1

**Figure 6.**
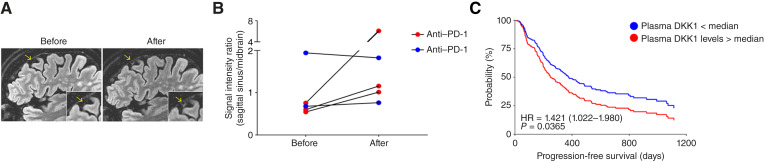
Radiologic and quantitative MRI findings reveal changes related to the BBB in human participants. **A,** Representative images of the 3D-FLAIR MRI of the brain of a patient with small cell lung carcinoma before and after receiving five cycles of anti–PD-1 treatment. Arrows point to the enhanced Gd intensity in the area surrounding the venous sinuses of the anti-PD-1–treated patient. **B,** The signal intensity ratio of Gd in the 3D-FLAIR MRI of the brain of patients with lung cancer before and after receiving anti–PD-1 or anti–PD-L1 treatment is plotted (*n* = 5 patients). **C,** A Kaplan–Meier plot depicting progression-free survival in patients with NSCLC stratified by post–anti-PD-1 plasma DKK1 levels above or below the median (*n* = 246). Cox proportional hazards regression demonstrated an HR of 1.421 (95% CI, 1.022–1.980; *P* = 0.0365).

Based on the preclinical observations, we next assessed the potential translation of these preclinical findings. In a historical cohort study of patients with NSCLC (*n* = 22), we retrospectively analyzed MRI images of the cohort at T1 before and after injection of gadolinium at baseline and 3 to 12 months after anti–PD-1 therapy. We observed increased signal intensity in the region of the sinus confluence after treatment in 14 of 22 patients (Supplementary Table S4). To validate these preliminary findings more comprehensively, we next conducted a prospective longitudinal trial in a small cohort of patients with BrM-free small cell or NSCLC receiving anti–PD-1 or anti–PD-L1 (Supplementary Table S5). We incorporated a 3D isotropic contrast-enhanced T2 fluid-attenuated inversion recovery (3D-FLAIR) protocol (34) into the standard imaging sequence to capture subtle parenchymal and meningeal enhancement while minimizing acquisition time compared with DCE-MRI. Our findings revealed substantial radiologic changes between pre- and posttreatment MRI scans, characterized by new or intensified gadolinium enhancement in patients receiving anti–PD-1. Notably, these changes were observed in areas surrounding the venous sinuses and CSF spaces in the meninges (Fig. 6A and B; Supplementary Table S5), similar to those found in mice. Consistent with our experimental data, these changes occurred exclusively in anti-PD-1–treated patients and were quantitatively confirmed, whereas anti-PD-L1–treated patients showed no comparable alterations (Fig. 6A and B; Supplementary Table S5).

### Plasma DKK1 Levels Correlate with BrM in Patients with NSCLC

Following the observed association between anti-PD-1–induced DKK1 upregulation and BBB disruption, we assessed whether plasma DKK1 dynamics correlate with BrM incidence in a cohort of patients with NSCLC. We analyzed a published dataset of plasma profiles from patients with NSCLC collected before and after initial immunotherapy (35). Only patients receiving anti–PD-1 monotherapy or anti–PD-1 + anti-CTLA4 (*n* = 246) were included; PD-L1–treated patients were excluded due to low numbers (*n* = 8). We stratified patients by posttreatment DKK1 fold change, defining significant upregulation as a >1.5-fold increase. Among all patients, those with a >1.5-fold increase in plasma DKK1 levels had a higher BrM frequency than those with ≤1.5-fold changes (39.4% vs. 24.5%), without statistical significance. To explore whether this relationship was affected by treatment response, we stratified the cohort into responders (*n* = 142) and nonresponders (*n* = 144). Among nonresponders, those with a >1.5-fold DKK1 increase had a 20% higher incidence of BrM than those with marginal DKK1 changes (*P* < 0.05). In contrast, among responders, BrM incidence was not significantly different between the two groups (33.3% vs. 26.4%; Supplementary Table S6). We observed a similar pattern when stratifying based on survival. Specifically, patients who died during follow-up, a substantial posttreatment increase in DKK1 was associated with a 23% higher BrM frequency (*P* < 0.05). However, among surviving patients, elevated DKK1 levels were not significantly linked to BrM incidence (33.3% vs. 25.0%; Supplementary Table S6). Notably, in the full cohort, elevated post–anti-PD-1 plasma DKK1 levels (above median) were significantly associated with shorter progression-free survival [hazard ratio (HR) = 1.421; 95% confidence interval (CI), 1.022–1.980; *P* = 0.0365], as determined by Cox proportional hazards analysis (Fig. 6C). Overall, these findings indicate that post–anti-PD-1 DKK1 upregulation correlates with an adverse clinical response in NSCLC, accompanied by a higher incidence of BrM.

To further strengthen these clinical findings, we asked under what circumstances host-mediated BBB opening, which may support cytotoxic T-cell entry and enhance anti–PD-1 activity, can become detrimental. Specifically, we investigated whether anti-PD-1–driven host responses alone promote intracranial progression or if outcomes are determined by tumor-intrinsic resistance. To this end, we employed a dual-site implantation model (Supplementary Fig. S15A, for illustration) using the parental LLC cell line, which is intrinsically resistant to immunotherapy, and a previously generated immunotherapy-sensitive mutagenized subline (16). The mutagenized subline was derived by chemically inducing mutations (see Supplemental Methods S1) in parental LLC cells, followed by *in vivo* selection for anti–PD-1 responsiveness. Mice first developed subcutaneous tumors, and once the tumors reached ∼40 mm^3^, the same cell type was implanted intracranially. Anti–PD-1/ IgG treatment was initiated on the day of intracranial implantation and continued for 1 week. As expected, anti–PD-1 did not control either subcutaneous or intracranial tumor growth in mice bearing the resistant parental line. In contrast, the mutagenized subline showed marked suppression of both subcutaneous and intracranial tumors although intracranial responses remained heterogeneous (Supplementary Fig. S15B and S15C). These findings indicate that host responses to anti–PD-1 alone are insufficient to drive intracranial progression and that intracranial outcomes are primarily governed by tumor-intrinsic sensitivity to PD-1 blockade. Flow cytometry of mutagenized intracranial tumors revealed increased CD8^+^ T- and B-cell infiltration following anti–PD-1, whereas CD4^+^ T cells, NK cells, DCs, G-MDSCs, and macrophages remained unchanged, indicating a shift toward a more active antitumor immune microenvironment (Supplementary Fig. S15D–S15L). Notably, anti–PD-1 treatment also increased M-MDSCs in mutagenized intracranial tumors (Supplementary Fig. S15J), a population linked to immune suppression and poor ICI outcomes in NSCLC (36). These findings suggest a complex interplay between host- and tumor-driven mechanisms in shaping intracranial growth.

### Single Dose Induction with Anti–PD-1 Increases the Efficacy of Cisplatin Treatment for BrM

**Figure 7.**
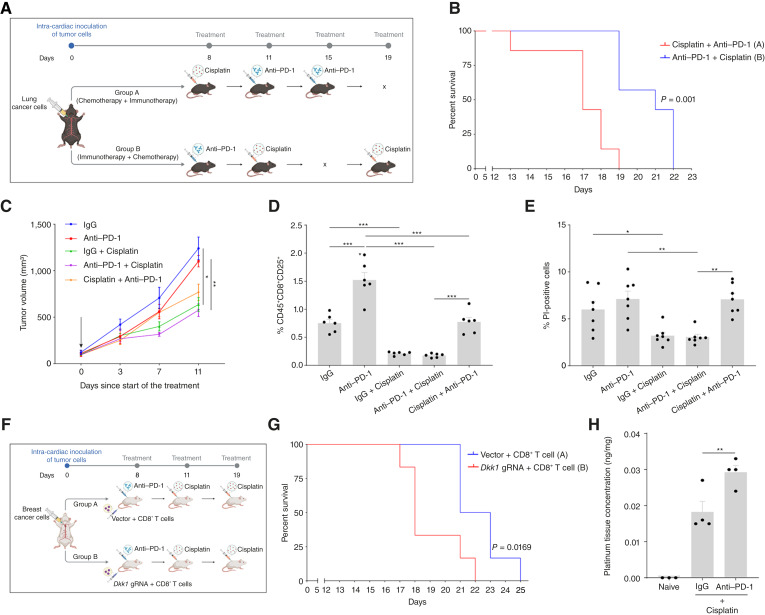
Induction with anti–PD-1 antibody prior to cisplatin chemotherapy increases the intracranial therapeutic efficacy. **A,** A schematic illustration depicting various treatment groups and their therapeutic regimens, evaluating the efficacy of anti–PD-1 and cisplatin-based combination therapy sequences on experimental BrM of anti-PD-1–resistant LLC cells. **B,** An experimental BrM assay was performed on 8-week-old C57BL/6 mice treated with anti–PD-1 and cisplatin chemotherapy in opposing sequences. A Kaplan–Meier survival curve is plotted (*n* = 6–7 mice/group). **C–E,** Eight-week-old C57BL/6 mice harboring subcutaneous LLC xenografts (∼100 mm^3^) were treated with IgG, anti–PD-1, IgG + cisplatin, anti–PD-1 + cisplatin, and the opposing sequence, cisplatin + anti–PD-1, and the therapeutic response was monitored. Tumor growth across various treatment groups is plotted (Arrow indicates start of the treatment; *n* = 4–5 mice/group; **C**). A bar graph showing the activation status of CD8^+^ T cells isolated from the spleens of these mice is plotted, as assessed by flow cytometry (**D**). A bar graph showing the percentage of dead cells [propidium iodide (PI)–positive] in a tumor cell killing assay, using CD8^+^ T cells isolated from the spleens of mice from the different conditions cocultured with LLC cells, as assessed by flow cytometry (**E**). **F** and **G,** Adoptive transfer of wild-type (Vector) and DKK1 KD [guide RNA (gRNA)] CD8^+^ T cells obtained from BALB/c mice was performed on 8-week-old SCID mice subjected to an experimental BrM assay using the 4T1 cancer cells. These mice were subsequently treated with a combination sequence of anti–PD-1 and cisplatin. An illustration depicting the experimental methodology for testing the effect of DKK1 KD on the therapeutic efficacy of the anti–PD-1 + cisplatin combination sequence is provided. Kaplan–Meier survival curve is plotted (*n* = 6 mice/group; **G**). **H,** Eight-week-old BALB/c mice, preconditioned with a single dose of IgG/anti–PD-1 or untreated (naïve), received cisplatin monotherapy 72 hours later. After 6 hours, the brains were harvested and prepared for the assessment of cisplatin concentration using LC/MS. A bar graph showing absolute quantification of cisplatin in the brains of BALB/c mice from various treatment groups is plotted (*n* = 3–4 mice/group). Significance was assessed by means of the log-rank test for **B** and **G**, two-way ANOVA for **C**, and one-way ANOVA for **D**, **E**, and **H**. *, *P* < 0.05; **, *P* < 0.01; ***, *P* < 0.001. [**A,** Created in BioRender. Raviv, Z. (2026) https://BioRender.com/rezdwrc; **F,** Created in BioRender. Raviv, Z. (2026) https://BioRender.com/oo7uvma.]

Opening of the BBB following anti–PD-1 treatment paves the way for enhanced efficacy of routine systemic cytotoxic therapies that show poor intracranial penetration in the treatment of BrM (37). To test this, we first evaluated whether chemotherapeutic agents themselves disrupt BBB integrity. The EB assay revealed that cisplatin and paclitaxel do not compromise BBB function at 24 or 72 hours after treatment (Supplementary Fig. S16). We next assessed whether anti-PD-1–induced BBB opening could sensitize BrM to chemotherapy using an experimental BrM model with LLC-luc cells, chosen for their complete resistance to anti–PD-1 (Supplementary Fig. S15B, parental LLC). Eight days after intracardiac inoculation of LLC-luc cells, animals were randomized into two treatment arms receiving combination therapy in opposite sequences: (i) anti–PD-1 followed by cisplatin or (ii) cisplatin followed by anti–PD-1, as illustrated in Fig. 7A. Mice receiving chemotherapy after anti–PD-1 induction had significantly improved survival compared with those treated with the reverse sequence (*P* < 0.01; Fig. 7B). To test whether this benefit resulted from enhanced CD8^+^ T-cell activity primed by anti–PD-1, we used a subcutaneous LLC model and analyzed T-cell responses after treatment. Although anti–PD-1 followed by cisplatin demonstrated better tumor control *in vivo* (Fig. 7C), CD8^+^ T cells isolated from these mice exhibited lower activation and reduced cytotoxicity (Fig. 7D and E) compared with the reverse sequence. These findings suggest that the therapeutic advantage arises primarily from enhanced drug delivery across the BBB rather than from augmented T-cell function, which may have been diminished by cisplatin exposure.

Given the central role of DKK1 in mediating anti-PD-1–induced BBB disruption, we next tested whether its depletion compromises the observed synergy. Using a *Dkk1*-KD adoptive transfer strategy, we introduced wild-type or *Dkk1*-KD CD8^+^ T cells into SCID mice bearing 4T1-BrM, an anti-PD-1–resistant model compatible with the BALB/c background (Fig. 7F). Mice receiving wild-type CD8^+^ T cells showed significantly improved survival following anti–PD-1 and cisplatin combination therapy, whereas those receiving *Dkk1*-KD CD8^+^ T cells did not (Fig. 7G). Finally, liquid chromatography/mass spectrometry (LC/MS) quantification of cisplatin confirmed increased drug accumulation in the brains of mice pretreated with anti–PD-1, relative to isotype control or cisplatin monotherapy (Fig. 7H). Together, these data establish that anti–PD-1 priming enhances the intracranial delivery and therapeutic efficacy of chemotherapy via DKK1-mediated BBB modulation, offering a rational strategy to treat immunotherapy-resistant BrM.

## Discussion

ICIs have shown clinical benefit in patients with BrM, including intracranial responses and, in some cases, prolonged survival. Monotherapy with anti–PD-1 yields durable responses in approximately 15% to 20% of patients with asymptomatic BrM, as demonstrated in melanoma cohorts (38, 39). However, response heterogeneity, ranging from complete response to partial response and even progressive disease, remains a significant clinical challenge. A recent study reports hyperprogression in up to 20% of BrM cases following ICI therapy (39). Although the extended duration of systemic response may contribute to the increased detection of BrM (40), we speculate that its concomitant progression could be partially attributed to host protumorigenic effects induced by ICI therapy (15). In this study, we define a host-mediated mechanism whereby anti–PD-1 therapy induces DKK1 expression in activated CD8^+^ T cells, leading to BBB permeabilization and remodeling of the brain–immune interface, a context that may underlie divergent intracranial responses to immunotherapy.

To the best of our knowledge, this is the first study demonstrating the BBB-permeabilizing effects of CD8^+^ T cells following anti–PD-1 therapy and providing supporting clinical evidence. MRI-based longitudinal assessments revealed functional BBB changes in patients with lung cancer undergoing immunotherapy, specifically anti–PD-1 treatment. However, these results only demonstrate the feasibility of BBB opening in humans, as the patient cohort was small (*n* = 5), including two patients treated with anti–PD-L1 who did not show gadolinium enhancement after therapy. Although these observations were supported by retrospective MRI analysis of a historical cohort of 22 patients, larger studies are needed to validate this phenomenon and further characterize BBB dynamics in this context. Interestingly, the BBB opening, both in mice and humans, was more prominent in the area of venous sinuses, implicating the importance of this region in patients treated with immunotherapy and suggesting that further clinical studies should evaluate the locoregional spread of metastasis in this context.

Here, we demonstrate that anti-PD-1–mediated activation of CD8^+^ T cells augments secretory DKK1, leading to BBB opening. DKK1, a canonical target of the Wnt–β-catenin–TCF signaling pathway, plays a crucial role in maintaining BBB integrity by stabilizing tight junctions in brain endothelial cells (32, 41). This aligns with our *in vitro* data demonstrating that DKK1 destabilizes endothelial barrier function through impairment of the Wnt–β-catenin–junction protein axis. CD8^+^ T cells were identified as the principal source of DKK1, with PD-1 blockade augmenting its expression via β-catenin/TCF and FOXM1 signaling. The β-catenin/TCF pathway, dynamically regulated in mature T cells, promotes memory CD8^+^ T-cell formation (42), whereas FOXM1 activation characterizes early T-cell maturation (33). DKK1 expression may thus be initiated by FOXM1 during activation and sustained by β-catenin/TCF signaling during later stages of activation.

Although DKK1-driven BBB opening by activated CD8^+^ T cells may facilitate immune cell infiltration and enhance intracranial antitumor immunity, the association of elevated DKK1 with BrM risk introduces an important duality. In this study, the experimental BrM model was used solely as a functional readout of enforced cancer cell entry into the brain and does not recapitulate the full metastatic cascade, in which BBB disruption represents only one component among others, such as cancer cell extravasation, local microenvironment conditioning, and immune-mediated effects (43). Thus, our study does not propose that anti–PD-1 therapy directly promotes BrM progression or metastatic relapse; rather, it provides a mechanistic basis for the intracranial heterogeneity. Consistently, in our dual-site implantation model, anti-PD-1–treated mice exhibited distinct intracranial responses at large, reflecting the intrinsic sensitivity of cancer cells rather than host factors such as elevated DKK1 levels alone. Notably, heterogeneous intracranial responses were accompanied by an increase in M-MDSCs, known mediators of immune suppression. Previously, stromal-derived DKK1 has been implicated in MDSC recruitment and immunosuppression in lung cancer (44). Thus, anti-PD-1–induced BBB opening may facilitate the infiltration of effector immune cells, such as CD8^+^ T cells, into the brain parenchyma, thereby suppressing tumor growth. However, this benefit may not be universal. The same increase in BBB permeability that enables immune cell infiltration may also permit tumor-promoting mechanisms to emerge. Supporting this, our scRNA-seq analysis revealed that MDSCs undergo protumorigenic reprogramming following anti–PD-1 treatment, which may create a permissive tumor microenvironment. In the absence of a preexisting antitumor immune signature, BBB disruption may thus facilitate rather than prevent tumor progression, emphasizing the context-dependent nature of immunotherapy responses in the brain. Therefore, we speculate that DKK1-mediated BBB modulation may be context-dependent, potentially aiding immune infiltration in responders while contributing to immune suppression and BrM risk in nonresponders, which may involve the reprogramming of stromal cells, a fact that we have also observed in patients with NSCLC.

The efficacy of chemotherapy in the treatment of BrM is generally lower than that in extracranial sites due to insufficient BBB penetration. Nevertheless, chemotherapy has been acknowledged for its role in improving the efficacy of ICI by boosting the expression of neoantigens, inducing immunogenic cell death, increasing PD-L1 expression in the tumor microenvironment, and promoting T-cell response (45–47). Here, we demonstrate that the BBB-permeabilizing effects of anti–PD-1 therapy enhance the delivery of cisplatin when immunotherapy is administered prior to chemotherapy in mice. This sequence elicits a stronger intracranial response in anti-PD-1–resistant BrM and results in improved survival compared with the conventional sequence. Clinically, however, the sequence and timing may be less critical as PD-1 antibodies have a prolonged half-life of several weeks (48). Therefore, the BBB-opening effects of initial PD-1 blockade are likely to persist into subsequent treatment cycles, allowing better penetration of chemotherapy even when both agents are administered concurrently. We speculate that this approach, using anti–PD-1 therapy to induce BBB opening, may serve as a therapeutic window not only for cancer but also for other neurologic indications, facilitating improved drug delivery. Nonetheless, the clinical observation that first-line chemoimmunotherapy demonstrates superior intracranial efficacy compared with immunotherapy alone in patients with NSCLC and BrM aligns with our findings (49).

In summary, our study provides mechanistic insight into the heterogeneous intracranial progression observed after immunotherapy and proposes a potential strategy to improve chemotherapeutic response in nonresponding patients with BrM undergoing immunotherapy. These findings advance current efforts to optimize therapeutic management in this challenging setting.

## Methods

### Cell Lines and Culture Conditions

Murine EMT6 (cat. #CRL-2755; RRID:CVCL_1923), 4T1 (cat. #CRL-2539; RRID:CVCL_0125) mammary carcinoma, and LLC (cat. #CRL-1642; RRID:CVCL_4358) cell lines were purchased from the ATCC. The murine brain endothelial b.End5 cell line was purchased from Sigma (cat. #96091930; RRID:CVCL_2252). All cells were cultured in high glucose DMEM (cat. #D5796) supplemented with 10% FBS (Gibco; cat. #10270-106), 1% L-glutamine (cat. #03-020-1B), 1% sodium pyruvate (cat. #S8636), and 1% penicillin–streptomycin (cat. #03-034-1B; Biological Industries) at 37°C in less than 5% CO_2_ conditions. The culture medium of the b.End5 cell line was additionally supplemented with 1% nonessential amino acids. The cells were used within 6 months of thawing from original vials and were routinely tested to be mycoplasma-free using the Mycoplasma PCR Detection Kit (ScienCell Research Laboratories).

### Animal Studies, Treatments, and Tumor Models

All animal experiments were approved by the Technion Institutional Animal Care and Use Committee and complied with ethics regulations. Female BALB/c (cat. #162), C57BL/6 (cat. #057), and SCID (cat. #182) mice (8 weeks of age) were purchased from Envigo. All mice were maintained under specific pathogen-free conditions in the animal facility and were housed in a temperature- and humidity-controlled environment in the animal facility, maintained under a 12-hour light–dark cycle. *Ad libitum* rodent chow and water were provided. All mice were habituated for a minimum of 1 week before the start of the experiment. The tumor models and treatments used in this study are described in detail in Supplementary data online.

### Cellular Experimental Procedures

All cellular experimental procedures, including cell mutagenesis, single-cell suspension, migration, conditioned medium generation, CD8^+^ T-cell activation, and cell killing, are described in detail in SDO.

### Plasma and Protein Experimental Procedures

All plasma and protein-associated experimental procedures, including ELISAs, arrays, and protein depletion, are described in detail in SDO.

### scRNA-Seq and Analysis

For single-cell sequencing, the single-cell suspension of brain tissue was prepared as described above, scRNA-seq libraries were prepared, and analysis was conducted as described previously (16). Detailed information is provided in SDO.

### Flow Cytometry Acquisition and Analysis

Immune cell populations analyzed through flow cytometry using surface markers indicated in Supplementary Table S7 are provided in SDO.

### EB Assay

The EB experimental procedure is described in detail in SDO.

### Immunostaining and Image Analysis

Freshly harvested brain tissues or T cells were immunostained for different markers as indicated in the text. For detailed experimental methods, see SDO.

### DCE-MRI in Mice

To assess the BBB function in mice, nine tesla DCE-MRI was performed. According to body weight, 50 to 70 µL of gadolinium–diethylenetriamine penta-acetic acid (Gd-DTPA) was injected via the tail vein into 8-week-old female BALB/c mice. The mice were either non–tumor-bearing or bearing orthotopic EMT6 breast tumors (∼200 mm^3^) and were treated with IgG, anti–PD-1, or left untreated. MRI of the brain was performed (i) prior to and (ii) at several time points after intravenous injection of Gd-DTPA. The protocol included T1w images, FLASH, 100 × 100 μm in plane resolution, 500 μm slice thickness, and 31 slices (TR/TE = 15/4.7 ms, 15° pulse). Analysis of MRI images: The volume of interest (VOI) was drawn in the area of the venous sinuses before and after Gd injection and repeated at all time points. Gd enhancement over time was calculated as the ratio of the pre-contrast VOI (first acquisition at *t* = 0, prior to Gd injection) to the post-contrast VOI at each time point. To visualize metastatic lesions, additional anatomic T2-weighted brain images were acquired using a rapid acquisition with relaxation enhancement sequence (RARE), at 500 μm slice thickness, 23 to 27 slices, and 100 μm in-plane resolution with repetition time (TR) = 3,500 ms, echo time (TE) = 36 ms, RARE factor = 12, field of view = 1.92 × 1.92 mm^2^, matrix size = 192 × 192, and number of averages = 2, along with an anatomic scan. Total scan time = ∼2 minutes.

### MRI Acquisition and Retrospective Analysis of Historical Cohort

The detailed description of MRI imaging and analysis of the retrospective study on patients with lung cancer (*n* = 22) is provided in SDO.

### 3D-FLAIR MRI in Patients with Lung Cancer

This study protocol was approved by the Institutional Review Board of Rambam Healthcare Campus (no. 0080-23-RMB). Patients diagnosed with NSCLC or small cell lung cancer (*n* = 5) were prospectively recruited for this study. Written informed consent was obtained from all patients prior to their involvement in the study. The inclusion criteria were exclusion of BrM, no history of neurologic disease, and treatment with immunotherapy only (anti–PD-1 or anti–PD-L1). Although the first MRI was performed before the administration of immunotherapy, subsequent imaging was carried out after 4–5 cycles of immunotherapy. The contrast agent used was Gd-DTPA. The sequence was adapted from a previously published protocol (34). The 3T MRI images were acquired using 3D-FLAIR sequences with a 1 mm slice thickness both before and after Gd administration. A sagittal 3D-FLAIR was captured using a fast spin echo sequence with inversion recovery preparation and variable refocusing flip angles (TR/TE/inversion time = 6,000/128/1,870 ms; 1 mm slice thickness). Reformatted images in axial and coronal planes were also obtained. Additionally, pre- and post-Gd T1-weighted imaging was performed using two pulse sequences: 3D T1 ultrafast gradient echo (TR/TE/inversion time = 9.18/3.68/450 ms, 1 mm slice thickness) and 3D T1 turbo spin echo (TR/TE = 600/12.98 ms, 1 mm slice thickness). All MRI scans were assessed by an experienced radiologist who was blinded to the study. The clinicopathologic characteristics and radiologic findings are shown in Supplementary Table S5.

### Molecular Experimental Procedures

All molecular experimental procedures, including PCR, gene modification, and cell signaling assays, along with the list of primers (Supplementary Table S8), are described in detail in SDO.

### Estimation of Cisplatin Using LC/MS

Estimation of cisplatin using LC/MS is described in detail in SDO.

### Statistical Analysis

All statistical tests were performed in GraphPad Prism version 10.6.1 (RRID:SCR_002798) and R version 4.1.0 software (RRID:SCR_001905). All experiments were performed in a randomized manner. Data are presented as mean ± SEM. In all *in vitro* experiments, at least three biological repeats were performed. For *in vivo* studies, the number of mice per group is indicated in the text, usually *n* = 4–7 mice/group, in order to reach statistical power considering a Gaussian distribution. Tumor growth was statistically compared using two-way analysis of variance (ANOVA). For *in vitro* studies, statistical significance within groups was assessed using one-way ANOVA, followed by Tukey *post hoc* test (when comparing more than two groups). For experiments involving only two groups for comparison, the Student *t* test was used to assess statistical significance. The frequency of metastasis was compared in mice using Fisher’s exact test and in humans using the χ^2^ test. Survival was analyzed using the Kaplan–Meier method, and differences between groups were compared by the log-rank test. Univariate Cox proportional hazards regression was performed to estimate HR and 95% CIs for the association between post–anti-PD-1 plasma DKK1 levels and progression-free survival. The significance of cell subtype proportion in the scRNA-seq data was assessed using a bias-corrected and accelerated bootstrap method for independent two-sample comparisons, followed by one-sided hypothesis testing, and conducted using wBoot (version 1.0.3). The Wilcoxon rank-sum test was used to analyze all single-cell differential gene expressions for the identified cell subsets. *P* values are represented as follows: *, *P* < 0.05; **, *P* < 0.01; and ***, *P* < 0.001.

## Supplementary Material

Supplementary Methods 1 Supplementary methods assocaited with the manuscript

Supplementary Table 1 List of genes used for supervised clustering

Supplementary Table 2 List of receptor-ligand interactions.

Supplementary Table 3 Log2-fold change in the levels of circulating plasma proteins in BALB/c mice treated with anti-PD1.

Supplementary Table 4 Clinicopathological features of patients of retrospective study

Supplementary Table 5 Clinicopathological features of patients of prospective study

Supplementary Table 6 Association of longitudinal increase in plasma DKK1 levels with brain metastasis

Supplementary Table 7 Cell surface markers analyzed using flow cytometry.

Supplementary Table 8 List of primers sequences used for qPCR analysis of specific genes

Supplementary Figure 1 Dot plot of cell populations identified in scRNA-seq data

Supplementary Figure 2 Gating strategy for flow cytometry analysis

Supplementary Figure 3 Validation of scRNA-seq data using flow cytometry

Supplementary Figure 4 Anti-PD1 therapy induces genetic alterations in myeloid-derived suppressor cells within the brain

Supplementary Figure 5 DCE-MRI assessment demonstrates alterations in the blood-brain barrier of anti-PD1 treated mice

Supplementary Figure 6 Assessment of BBB permeability in response to different immune checkpoint inhibitors

Supplementary Figure 7 Mice primed with anti-PD1 therapy exhibited increased brain metastasis of lung cancer cells

Supplementary Figure 8 DKK1 in plasma from anti-PD1 treated mice disrupts the integrity of endothelial cells

Supplementary Figure 9 The effect of DKK1 on β-catenin–regulated tight junction proteins in endothelial cell

Supplementary Figure 10 Transfusing plasma from anti-PD1-treated BALB/c mice into SCID mice leads to BBB opening

Supplementary Figure 11 Dkk1 knockdown in CD8+ T cells

Supplementary Figure 12 Flow cytometry analysis of PD-L1 and PD-L2

Supplementary Figure 13 The percentage of DKK1+ on active CD8+ T cells under different ICI-treated conditions

Supplementary Figure 14 Effect of Ctnnb1 knockdown on Dkk1 expression in CD8⁺ T cells

Supplementary Figure 15 Effect of anti-PD1 therapy on the intracranial progression of sensitive vs. resistant lung cancer cells.

Supplementary Figure 16 Chemotherapy does not alter the permeability of the blood-brain barrier

## Data Availability

The scRNA-seq dataset generated in the current study is publicly available in the Code Ocean repository at https://codeocean.com/capsule/4833035/tree/v1.
