# Supplementary material for: PD-1 Blockade–Induced DKK1 Expression by CD8+ T Cells Promotes Blood–Brain Barrier Permeabilization
Source: Cancer Discov. 2026 Jan 13;16(5):976–92. doi: 10.1158/2159-8290.CD-25-1222 (PMC13133603; doi:10.1158/2159-8290.CD-25-1222)
Supplement: Supplementary Methods 1 — Supplementary methods assocaited with the manuscript [file cd-25-1222_supplementary_methods_1_suppsm1.pdf]

SUPPLEMENTARY DATA ONLINE

Deo et. al.,

SUPPLEMENTARY MATERIALS AND METHODS

Cell line mutagenesis

Cell line mutagenesis was performed as previously described (1). Briefly, the ICI-resistant luciferase tagged parental LLC cell line was exposed to 1-methyl-3-nitro-1-nitrosoguanidine (MNNG; Apollo Scientific, UK; Cat#OR301388) for 2 hours. Subsequently, cells were washed with PBS, replenished with growth medium, and allowed to recover for 5 days to generate a multiclonal pool of mutagenized cells. This procedure was repeated by a second round of mutagenesis using 99% ethyl methanesulfonate (EMS; Thermo Fisher Scientific, USA; Cat#205260100), also for 2 hours. The resulting cell population was subsequently validated *in vivo* for responsiveness to anti-PD1 therapy.

Tumor models

Subcutaneous lung cancer xenograft model: The subcutaneous tumor xenograft model of lung cancer was developed by injecting 0.5×10^6 LLC cancer cells resuspended in 100 µl of Hank's balanced salt solution (HBSS) (Sigma, Rehovot, Israel;Cat#H6648) subcutaneously into the flank of 8 weeks old female C57BL/6 mice. The tumor volumes were measured twice a week with a caliper and calculated using the formula $0.5 \times \text{Length} \times (\text{Width})^2$. The drug treatment was initiated once the tumor size reached ~100 mm^3 .

Intracranial experimental brain metastasis model: Intracranial implantation was performed as previously described(2). In brief, 8-week-old male C57BL/6 mice were anesthetized with isoflurane and secured in a Kopf stereotaxic frame. A total of 25,000 parental or mutagenized LLC cells suspended in 5 µl of PBS were injected into the brain. The progression of brain metastases was monitored using bioluminescence imaging.

Orthotropic breast cancer model: An orthotopic breast cancer mouse model was developed by injecting 0.5×10^6 EMT6 cancer cells resuspended in 50 µl of Hank's balanced salt solution (HBSS) in the mammary fat pad of 8 weeks old female BALB/c mice. The tumor volumes were measured twice a week with callipers and calculated using the formula $0.5 \times \text{Length} \times (\text{Width})^2$.

Intracardiac experimental brain metastasis model: An experimental brain metastasis mouse model was established as previously published(3). Briefly, 0.5×10^6 EMT6, LLC cells stably expressing firefly luciferase (EMT6-luc and LLC-luc, respectively) and 4T1 cells in 100 µl of HBSS through the left ventricle guided through micro ultra-sound (VisualSonics Vevo® 3100 Imaging System; RRID:SCR_022152) using a 30G long needle in 8-week-old female BALB/c, C57BL/6 and SCID mice, respectively. The development

of brain metastasis was monitored through bioluminescence imaging, using IVIS Lumina X5 (PerkinElmer, US), for up to 4 weeks following which the experiment was terminated. Throughout the experiment, the mice were monitored for neurological symptoms. In the therapeutic efficacy experiments (Fig. 7), overall survival was defined as the duration from implantation (Day 0) to death.

For animal experiments involving adoptive transfer of $CD8^+$ T cells, single-cell suspensions were prepared from spleens harvested from EMT6 or 4T1 tumor bearing BALB/c mice. The protocol for isolation of $CD8^+$ T cells is described elsewhere. Subsequently, the collected cells were intravenously injected (5×10^6 per mouse per injection) into 8-week-old naive SCID mice (Envigo).

In all animal experiments, when mice did not develop tumors or displayed unexpected clinical symptoms, they were removed from the analysis.

Plasma antibody and protein depletion

For antibody depletion, plasma samples were incubated with Protein G Sepharose® beads (50 µl beads/ml of plasma) (Abcam, UK; Cat#ab193259) at 4°C for 2 hours on a shaker followed by centrifugation at 2000 × g for 10 min to remove the beads. For the depletion of specific plasma proteins, plasma samples were incubated with 1 µg of anti-DKK1 (R&D Systems, USA; Cat#AF1096; RRID:AB_354597), anti-Lipocalin-2 (R&D Systems, USA; Cat#AF1857; RRID:AB_355022), or anti-CCL4 (Abcam, UK; Cat# ab45690-100; RRID:AB_776129) antibodies overnight at 4°C which were subsequently pulled down using Sepharose beads.

Brain single cell suspension

Eight-week-old BALB/c mice were implanted with EMT6 cells to develop orthotopic breast tumors as mentioned above. Once the tumor size reached 200 mm^3, the mice were treated with isotype IgG or anti-PD1 antibodies. After one week, the mice were perfused with ice cold PBS, brain tissues were harvested, and a single-cell suspension was prepared. Briefly, the brain tissues were gently minced using a sterile scalpel to yield small pieces that were subsequently digested using dispase and collagenase at 37°C for 15 min. Next, the tissue homogenate was passed through sterile 70-µ cell strainer followed by Percol gradient centrifugation. The cells thus harvested were collected, washed, and resuspended in 0.04% BSA in PBS to a concentration of 1000 cells/µl for further processing.

Single cell RNA sequencing and analysis

For single cell sequencing, the single cell suspension of brain tissue was prepared as described above and scRNA-seq libraries were prepared according to 10x manufacture protocol (Chromium Next GEM Single Cell 3' Library & Gel Bead Kit v3.1, PN-1000121) using 20,000 input cells per sample (aiming for ~10,000

target cells per sample). Single cell separation was performed using the Chromium Next GEM Chip G Single Cell Kit (PN-1000120). The RNA-seq data was generated on Illumina NextSeq2000, P2 100 cycles (Read1-28; Read2-90; Index1-10; Index2-10) (Illumina, Cat#20046811). Read mapping and cell calling was performed using 10X Genomics software - CellRanger [v7.1.0] using the "cellranger count" function. The function inputs were samples FASTQ files. The 10x specific genome reference Mouse (mm10) was used as a reference for the alignment of the reads. The function was run with "--include-introns" flags to include intronic reads. After generating the data, the results showed that in both samples more than 5,000 cells were called and the median number of genes detected per cell was about 1,500.

The Seurat pipeline [v4.3.0] was used for downstream analysis (4). Data were read into R [v4.2.2] as a counts matrix and genes expressed in less than 10 cells were discarded. We set gene expression cut-offs at minimum of 500 and a maximum cut-off of 6,000 genes per cell. Additionally, cells with a percentage of total reads that aligned to the mitochondrial genome greater than 10% were removed as likely dead cells. We also utilized scDblFinder [v1.12.0] to remove doublets (5). We normalized and scaled the data using the "SCTransform"[v2] function, selected 3000 variable features, and linearly regressed out any remaining influence of the mitochondrial genome on downstream analyses.

Dimensionality reduction was performed with principal component analysis followed by tSNE projection. Unsupervised clustering using the shared nearest neighbor (SNN) and the first 17 principal components, was performed using the Seurat functions "FindNeighbors" and "FindClusters" (resolution=0.3). For cell type identification, we generated a gene set of different populations markers and employed the Semi-supervised Category Identification and Assignment (SCINA) method (6). Considering the top differentially expressed genes between the clusters, we identified 21 different cell types based on the following sources: Mouse Brain Atlas (mousebrain.org); CellMarker (xbio.top); Azimuth (hubmapconsortium.org). MDSCs were characterized based on their signatures as previously described (7). Finally, cells with an overall low signature expression were defined as "unknown" (default mode) and filtered out from the analysis.

Cellular neighborhoods displaying differential abundance (DA) between aPD1 and IgG were defined by DASeq [v1.0.0](8), using the principal components matrix after dimension reduction of the MDSC cluster. The CommPath package was utilized for the analysis of the pathway mediate cell-cell communication chain, as previously described (9). Briefly, we processed each sample separately to create CommPath objects. The normalized matrix from Seurat (RNA assay) was used as input.

We identified significantly highly expressed ligands and receptors (logFC > 0.5 & p-value > 0.01) for each cell type and the signaling pathways containing the upregulated ligands or receptors using GO pathways. Finally, we assessed the differentially activated pathways and the communication chains of endothelial cells between IgG- and anti-PD1-treated samples.

Drug and plasma treatments

Anti-rat antibodies—including anti-PD1 (clone RMP1-14) (BioXCell; Cat#BE0146; RRID:AB_10949053 or IchorBio; Cat#ICH1132, RRID:AB_2921498), anti-PD1 (clone 29F.1A12™) (BioXCell; Cat#BE0273; RRID:AB_2687796), anti-PDL1 (clone 10F.9G2) (IchorBio; Cat#ICH1086; RRID:AB_2921473), anti-PDL2 (clone TY25) (IchorBio; Cat# ICH1139), anti-CTLA4 (clone 9H10) (IchorBio; Cat#ICH1084; RRID:AB_2921471), and isotype control rat IgG (BioXCell; Cat# BE0089; RRID: AB_1107769 or IchorBio; Cat#ICH2244; RRID:AB_2921379)—as well as murinized antibodies such as anti-PD1 (RMP1-14-CP151) (BioXCell; Cat#CP151; RRID:AB_2927525) and murinized isotype control IgG (BioXCell; Cat#CP150; RRID:AB_2927524), were administered intraperitoneally to mice at a dose of 5 mg/kg, three times per week with 48-hour intervals between doses. Mice were sacrificed 24 hours after the last treatment. Lipopolysaccharide (LPS) (Sigma, Rehovot, Israel; Cat#L2880) was delivered to mice intraperitoneally as a single dose (3 mg/kg) 24 hours before the assay. Cisplatin (Pharmachemie B.V., Netherlands) and paclitaxel (Teva Pharmaceuticals, USA) were administered intraperitoneally at the maximum tolerated dose of 6 mg/kg and 25 mg/kg, respectively. In the efficacy study (Fig. 6A), chemotherapy alone group received two doses of cisplatin at an interval of 7 days. (For all *in vitro* and *in vivo* experiments involving anti-PD1 treatment, the RMP1-14 clone was used unless otherwise specified). Plasma was collected and pooled from IgG- (hereon termed as IgG-plasma) and anti-PD1-treated (hereon termed as anti-PD1-plasma) non-tumor bearing BALB/c or C57BL/6 mice at the end of the treatment cycles described above. Following antibody depletion, outlined below, plasma was intraperitoneally injected (100 µl/dose) in non-tumor bearing BALB/c orC57BL/6 mice thrice a week at an interval of 48 hours. For in vitro assays assessing the effect of DKK1, cells were treated with recombinant DKK1 (R&D Systems, USA; Cat#5897-DK-010) at a concentration of 100 ng/mL for the indicated durations.

Flow cytometry acquisition and analysis

Immune cell populations identified in scRNA-seq analysis were validated through immunostaining of single cell suspensions prepared from mouse brain tissues. This validation involved the use of specific surface markers to identify different immune cell types as indicated in Table S7. All antibodies were purchased from BioLegend, USA or BD Biosciences, USA [APC/Cyanine7 anti-human CD45 Antibody (HI30), Cat#304014, RRID:AB_314402; Brilliant Violet 605_ anti-mouse/human CD45R/B220 Antibody (RA3-6B2), Cat#103243, RRID:AB_2563312; Brilliant Violet 510_ anti-mouse CD4 Antibody (GK1.5), Cat#100449, RRID: AB_2564587; APC/Cyanine7 anti-mouse CD8a Antibody (53-6.7), Cat#100714, RRID:AB_312753; APC anti-mouse CD25 Antibody (PC61), Cat#102012, RRID:AB_312860; APC/Cyanine7 anti-mouse CD11c Antibody (N418), Cat#117323, RRID:AB_830646; Brilliant Violet 605_ anti-human CD11b Antibody (ICRF44), Cat#301332, RRID:AB_2562020; PE/Cyanine7 anti-mouse

CD335 (NKp46) Antibody (29A1.4), Cat#137617, RRID:AB_11218594; BD OptiBuild™ BV711 Hamster Anti-Mouse CD49b (HMα2), Cat#740704, RRID:AB_2740388; PE/Cyanine7 anti-mouse Ly-6C (HK1.4), Cat# 128017, RRID:AB_1732093; Brilliant Violet 510_ anti-mouse Ly-6G (1A8), Cat#127633, RRID: AB_2562937; PE anti-mouse F4/80 Antibody (BM8), Cat# 123110, RRID: AB_893498] and were used in accordance with the manufacturer's instructions. The internal staining of granzyme B and DKK1 was performed using Fix/Perm Buffer Set (BioLegend, USA: Cat# 421401). The samples were acquired using a BD LSRFortessa flow cytometer (RRID: SCR_018655) and analyzed with FlowJo V.10 software (FlowJo, Ashland, Oregon, USA).

Evans blue (EB) assay

Left ventricle administration: Eight-week-old BALB/c or SCID mice treated with anti-PD1, IgG, or LPS; mice were anesthetized using isoflurane and subsequently perfused with 200 µl of 0.5% Evans blue (EB) dye (Sigma, Rehovot, Israel; Cat#E2129 through intracardiac injection. Afterward, mice were decapitated to harvest whole brains. After capturing the images of the whole brains, a part of the tissue was embedded in the optimal cutting temperature compound (OCT) while the other part was chopped into small pieces, dried at 60°C for 3 hours and weighed followed by incubation with 200 µl formamide (Sigma, Rehovot, Israel; Cat# F9037) at room temperature for 72 hours to extract the dye. Subsequently, the solution was collected and absorbance was measured at 610 nm in triplicates/sample, using Infinite 200 PRO ELISA reader (Tecan, Switzerland). The extravasation of EB dye in each tissue was calculated by the average absorbance at 610 nm/gr of tissue.

Tail vein administration: Eight-week-old BALB/c mice treated with anti-PD1 or isotype IgG, were intravenously injected with 2% EB dye (4 ml/kg body weight) via the tail vein. After 4 hours, mice were anesthetized with isoflurane and perfused intracardially with sterile PBS until the outflow ran clear. Whole brains were harvested and homogenized in 1 ml of 50% (w/v) trichloroacetic acid (TCA) in PBS. Homogenates were centrifuged at 6000 × g for 20 minutes at 4°C, and the resulting supernatants were diluted 1:3 with 95% ethanol. Absorbance was measured at 610 nm in triplicate using the Infinite 200 PRO ELISA reader (Tecan, Switzerland). EB concentrations were quantified using a standard curve (4.096 ng–1000 ng/ml) prepared in the same solvent. Results were normalized to tissue weight and expressed as average absorbance per gr tissue.

Immunostaining and image analysis

Freshly harvested brain tissues were embedded in OCT, frozen at −80°C, and sectioned at 5µm onto Epredia™ SuperFrost Plus™ adhesion slides (Epredia, USA; Cat#110002564). For immunostaining, frozen sections were thawed at room temperature for 10 min and fixed with chilled acetone at −20°C for 15 min.

Sections were washed with PBS, permeabilized with 0.1% Triton X-100 for 5 min at room temperature, washed, and blocked with 10% goat serum (Biological Industries, Israel; Cat#04-009-1A) for 1 hour at room temperature. Sections were incubated overnight at 4°C with the following primary antibodies: anti-CD31 (BD Biosciences, USA; Cat#553370; RRID:AB_394816), anti-claudin-5 (Abcam, UK; Cat#ab131259; RRID:AB_11157940), and anti-occludin (GeneTex, USA; Cat#GTX114949; RRID: AB_11177242). After washing, sections were incubated with appropriate secondary antibodies (anti-rabbit and anti-rat) for 2 hours at room temperature and mounted with mounting medium.

For granzyme B–DKK1 colocalization studies, microscope coverslips (22×22 mm) were overlaid with a single-cell suspension of isolated $CD8^+$ T cells, and incubated for 2 hours at room temperature. Coverslips were then fixed with 10% formalin for 10 min at room temperature, permeabilized with 0.5% Triton X-100 for 10 min, and blocked with 10% goat serum for 1 hour at room temperature. Coverslips were incubated overnight at 4°C with anti-granzyme B (BioLegend, USA; Cat#372206; RRID:AB_2687030) and anti-DKK1 primary antibodies, washed, incubated with appropriate secondary antibodies for 2 hours at room temperature, and mounted.

For endothelial cell immunostaining, bEnd.5 brain endothelial cells grown as monolayers on coverslips were treated with PBS (control) or recombinant DKK1 (100 ng/ml for 24 hrs). Following incubation, the cells were fixed with 4% paraformaldehyde (PFA) for 15 min at room temperature, permeabilized with 0.1% Triton X-100 for 5 min at room temperature, and blocked with 10% goat serum for 1 hour at room temperature. Cells were incubated overnight at 4°C with the following primary antibodies: anti-claudin-5 (Abcam, UK; Cat#ab131259; RRID:AB_11157940), anti-occludin anti-occludin (GeneTex, USA; Cat#GTX114949; RRID: AB_11177242), anti-VE-cadherin (RDI, USA; Cat#RDI-MCD144), anti-phospho-β-catenin (Ser45) (CST, USA; Cat#9564; RRID:AB_331150), and anti–β-catenin (Sigma, Rehovot, Israel; Cat#C2206; RRID:AB_476831). After washing, coverslips were incubated with appropriate secondary antibodies for 2 hours at room temperature and mounted with mounting medium.

A Zeiss LSM 710 confocal microscope was used for image acquisition, and images were analyzed using FIJI (ImageJ) (RRID: SCR_002285). Pearson's correlation coefficient for colocalization was quantified using BIOP's version of the JACoP (Just Another Colocalization Plugin) FIJI plugin.

Trans-endothelial cell migration assay

Boyden chamber 8.0 µm inserts (Falcon, USA; Cat#353097) pre-coated with rat tail collagen type I (Corning, USA; Cat#354236) were overlaid with b.End5 cells in complete medium and incubated at 37°C for 24 hours. After monolayer confluency was reached, the medium was replaced with fresh medium containing 5% IgG-, or anti-PD1-plasma and the inserts were incubated at 37°C for 48 hours. Next, EMT6 cells stably expressing GFP (EMT6-GFP) pre-treated with IgG- or ant-PD1-plasma were seeded on top of

this monolayer in incomplete medium, while complete medium was added to the lower chamber, and incubated at 37°C for 24 hours. For the LPS group, b.End5 monolayers grown on inserts were treated for 24 hours before adding untreated EMT6-GFP cells to the upper chamber. Similarly, to assess the effect of DKK1, b.End5 monolayers were treated with DKK1 for 48 hours prior to adding untreated EMT6-GFP cells. After incubation, the inserts were removed and washed, and the insides were scraped to remove the non-migrated fraction of cells. Images were acquired using LEICA DMi8 fluorescence inverted microscope and the migrated fraction was measured and represented as GFP intensity/field.

Plasma protein array

Plasma was obtained from tumor-free, 8-week-old BALB/c mice treated with IgG or anti-PD1 antibodies for one week as described elsewhere. The plasmas were pooled per group and applied to Proteome Profiler Mouse XL Cytokine Array chips (R&D Systems, USA; Cat#ARY028), in accordance with the manufacturer's instruction. The array chips were scanned and the signals corresponding to each factor in the array were quantified using the FIJI (ImageJ) software (RRID: SCR_002285). The alterations in protein levels were assessed by calculating the $\log_2$ fold change of the anti-PD1 to IgG ratio (Table S3).

Isolation of immune cells and preparation of conditioned medium

For preparation of the conditioned medium, a single cell suspension was prepared from spleens harvested from tumor bearing IgG- and anti-PD1-tretaed BALB/c mice. $CD4^+$ T (Cat#BLG-480006), $CD8^+$ T (Cat#BLG-480008), and B cells (Cat#BLG-480052) were isolated from single cell suspension of splenocytes using anti-CD8, CD4, and B220 antibodies, bound to magnetic beads (BioLegend, USA) using magnetic columns for isolation. Next, the cells were resuspended at a density of 2×10^6 cells/ml of complete RPMI supplemented with 1% L-glutamine, 1% sodium-pyruvate, and 1% penicillin–streptomycin. After incubation at 37°C for 24 hours the conditioned medium was collected.

ELISA

Plasma and conditioned media levels of Lipocalin-2 (LCN2) (Cat#DY1857-05), CCL4/MIP-1β (Cat#DY451-05), DKK1 (Cat#DY1765), and granzyme B (Cat#DY1865) were quantified using mouse DuoSet ELISA kits specific for each analyte (R&D Systems, USA), according to the manufacturer's instructions.

CRISPR mediated *Dkk1* and *Ctnnb1* knock-down in $CD8^+$ T cells

Dkk1 gRNA (Forward: 5'CACCGCTACCCTTGCGCTGAAGATG3'; Reverse: 5'AAACCATCTTCAGCGCAAGGGTAGC3') and *Ctnnb1* (Forward:

5'GACAAAACTGCTAAACGATG3'; Reverse: 5'CATCGTTTAGCAGTTTTGTC3') gRNA were cloned into lentiCRISPR v2 vector plasmid (Addgene plasmid#52961; RRID:Addgene_52961). Next, lentiviral particles were generated by co-transfecting HEK-293FT cells with packaging (psPAX2; Addgene plasmid#12260; RRID:Addgene_12260) and envelope (pMD2.G; Addgene plasmid#12259; RRID:Addgene_12259) plasmids together with lentiCRISPR v2 vector (control) or lentiCRISPR v2 vector containing DKK1 specific gRNA or CTNNB1 specific gRNA. The supernatant containing the viral particles was harvested 48-hours post-transfection, filtered through a 0.45-mm syringe filter followed by transduction into $CD8^+$ T cells isolated from the spleen as described elsewhere. Subsequently, the knockdown was confirmed from the conditioned media of small proportion of cells using ELISA for DKK1 and through real time PCR at the transcript level for DKK1 and CTNNB1, and the remaining cells were utilized for further processing.

***In vitro* activation of $CD8^+$ cells**

$CD8^+$ T cells were isolated from the spleens of non-tumor-bearing C57BL/6 mice and treated with phorbol 12-myristate 13-acetate (PMA) (Sigma, Rehovot, Israel; Cat#P8139) (10ng/ml) and ionomycin (Sigma, Rehovot, Israel; Cat#I0634) (10µg/ml) at 37°C for 24 hours. Following incubation, the cells were treated with in IgG, anti-PD1, anti-PDL1, and anti-CTLA4 antibodies (10µg/ml) at 37°C for 24 hours. Next, the cells were harvested for RNA isolation and subsequent gene expression studies.

Co-culture of $CD8^+$ T and LLC cells

$CD8^+$ T cells were isolated from the spleens of non-tumor-bearing C57BL/6 mice, chemically activated with PMA and ionomycin, and co-cultured with LLC cells (1:10 ratio) in the presence of IgG, anti-PD1, anti-PDL1, anti-PDL2, and anti-CTLA4 antibodies (10µg/ml) at 37°C for 24 hours. After incubation, the cancer cell-$CD8^+$ T cells were harvested and immunostained for CD45, CD8, GranzymeB, and DKK1 markers (Table S7) and activation was assessed using flow cytometry.

Quantitative reverse transcription PCR

Total RNA was extracted from cells using total RNA purification kit (Norgen Biotek Corp., Canada; Cat#35300) according to the manufacturer's instructions. Equivalent quantities of total RNA were reverse-transcribed with using the High-Capacity cDNA Reverse Transcription Kit (Thermo Fisher scientific, USA; Cat#AB-4374966). Complementary DNAs (cDNAs) were diluted and were analyzed by quantitative real-time PCR analysis (Applied Biosystems, StepOnePlus) using gene specific primers. The expression of each gene was normalized to *Gapdh* expression. List of primers sequences used for qPCR analysis of specific genes is in Table S8.

β-catenin/TCF and FOXM1 inhibition

$CD8^+$ T cells were isolated from the spleens of C57BL/6 mice and treated with PMA (10ng/ml) and Ionomycin (10μg/ml) at 37°C for 24 hours. Following incubation, the cells were treated with IgG and anti-PD1 (10μg/ml) along with DMSO (Sigma, Rehovot, Israel; Cat#D5879), β-catenin/TCF inhibitor: PKF118-310 (Merck, USA; Cat#219331) (1 μM) and FOXM1 inhibitor: FDI-6 (Selleck chemicals, USA; Cat#S9689) (30 μM) at 37°C for 24 hours. Next, cells were harvested for RNA isolation to assess *Dkk1* transcript levels by real-time PCR, while conditioned media was collected for the detection of secretory DKK1 by ELISA.

MRI acquisition and retrospective analysis of historical cohort

This retrospective study included NSCLC patients (n=22) undergoing treatment with anti-PD1. The study has been approved by the ethics committee of the Medical Faculty of Heidelberg University (approval No. S-145/2017), and all patients signed informed consent. Each patient underwent MRI prior to the initiation of therapy and again between 3 and 12 months after the first treatment cycle. Both MRI examinations were performed using the same1.5 Tesla scanner (Magnetom Aera; Siemens Medical Systems, Erlangen, Germany) at the Thorax Clinic at University of Heidelberg Medical Center, Germany. The imaging protocol remained consistent between baseline and follow-up scans, with no variation in repetition time (TR) or echo time (TE). Parameters for all axial T1-weighted MPRAGE sequences were as follows: Repetition time (TR): 2000 ms; Echo time (TE): 3.02 ms; Section thickness: 1.0 mm; Field of view: 250 × 250 mm. The contrast agent dose was standardized to 0.1 mmol per kilogram of the patient's body weight. The MRI images were analyzed using the picture archiving and communication system (PACS) and reviewed by a single investigator. The region of interest (ROI) was manually delineated within the superior sagittal sinus, directly above the confluence of sinuses, at the point of greatest diameter in the post-contrast baseline image. Peripheral areas were excluded. This ROI was then copied to the corresponding locations in all subsequent sequences. The difference in signal intensity between the baseline and follow-up MRI scans was calculated. The clinicopathological are shown in Table S4.

Tumor cell killing assay

LLC cells were seeded in a 48-well plate at a density of 50,000 cells per well, along with 5×10^5 $CD8^+$ T cells (1:10 ratio) isolated from LLC tumor-bearing mice from various treatment groups: IgG, anti-PD1, IgG+cisplatin, anti-PD1+cisplatin, and the opposite sequence, cisplatin+anti-PD1 for 24 hours. After incubation, the cancer cell-$CD8^+$ T cells were harvested and immunostained for CD8 and CD25 markers (Table S7). PI (500 nM) was also added to the cultures to label dead cells. The T cell activation and tumor killing effect was assessed using flow cytometry.

Estimation of cisplatin using liquid chromatography-mass spectrometry (LC/MS)

Eight-week old non-tumor bearing BALB/c mice were subjected to a single dose of either anti-PD1 or isotype IgG (5 mg/kg) for a duration of 72 hours, followed by an additional treatment with cisplatin chemotherapy or left untreated. The animals were sacrificed six hours after cisplatin treatment, and their brains were collected, weighed and snap frozen.

The sample preparation for LC/MS was carried out as described previously with some modifications (10). Frozen brain tissues were homogenized in sodium diethyldithiocarbamate (DDTC) (Merck, D3506) solution (1% DDTC in 0.1 M NaOH solution) (1ml/brain) at 4 C^0 in a Precellys 24 tissue homogenizer (Bertin Corp, P002391-P24T0-A.0) (Three cycles, 30 seconds each, 6000 rpm with a 30 second intermittent gap) following incubation at 40 °C for 30 min. Next, 2.5 mL of ethyl alcohol/xylene (1:1; v/v) was added to the mixture, vortex-mixed for 5 minutes and the tubes were centrifuged at 11,600g/15 min at 4 °C. Subsequently, the upper organic phase was collected, evaporated to dryness under nitrogen followed by reconstitution in 100 µl of acetonitrile/H2O (1:1; v/v). The tubes were vortexed for 15 minutes, centrifuged at 11,600g /15 min at 4 °C and the supernatant was transferred into HPLC glass vials (Agilent, 8010-0542) and stored at -80°C until LC-MS analysis. For absolute quantification, calibration curve samples were prepared at 0.5, 2.5, 5.0, 10.0, 50.0, 250.0 ng/mL by serial dilution from the stock solution prepared in acetonitrile (Merck, 100029) /H2O (1:1; v/v). To account for matrix effect, all calibration samples were diluted into dried down, untreated, brain tissues matrix that went through the same extraction and derivatization process. LC-MS metabolomics analysis was performed using Vanquish Flex™ ultra HPLC system coupled to Exploris 240™ Orbitrap Mass Spectrometer (Thermo Fisher Scientific) with a resolution of 120,000 at 200 mass/charge ratio (m/z). Electrospray ionization at positive mode (4000 v) was used across a mass range of 67 to 1000 m/z. RF% was set to 70. Sheath and Aux gas was set to 35 and 15 respectively (arbitrary units) with ion transfer tube at 300 ^{0}C and vaporizer temperature at 100 ^{0}C. HPLC setup consisted of Hypersil GOLD™ (100 mm x 2.1 mm, 3µm) (Thermo, 25003-102130), 5 µL of biological extracts were injected and the compounds were separated with mobile phase consisting of 10% water (mobile phase A) and 90% of mobile phase B composed of 0.1% acetic acid and 10mM ammonium acetate in 95:5 (Acetonitrile :Water). Flow rate and column temperature were maintained at 0.3 mL/min and 45 ^{0}C, respectively, for a total run time of 10 minutes. Cisplatin derivatives were detected using a mass accuracy below 5 ppm. Xcalibur (ThermoFisher Scientific) was used for data acquisition. Cisplatin compounds were identified using authentic standard that went through the same derivatization process.

REFERENCES

1. Benguigui M, Cooper TJ, Kalkar P, Schif-Zuck S, Halaban R, Bacchiocchi A*, et al.* Interferon-stimulated neutrophils as a predictor of immunotherapy response. Cancer Cell **2024**;42(2):253-65 e12
2. Levy A, Blacher E, Vaknine H, Lund FE, Stein R, Mayo L. CD38 deficiency in the tumor microenvironment attenuates glioma progression and modulates features of tumor-associated microglia/macrophages. Neuro Oncol **2012**;14(8):1037-49
3. Zhou H, Zhao D. Ultrasound imaging-guided intracardiac injection to develop a mouse model of breast cancer brain metastases followed by longitudinal MRI. J Vis Exp **2014**(85)
4. Stuart T, Butler A, Hoffman P, Hafemeister C, Papalexi E, Mauck WM, 3rd*, et al.* Comprehensive Integration of Single-Cell Data. Cell **2019**;177(7):1888-902 e21
5. Germain PL, Lun A, Garcia Meixide C, Macnair W, Robinson MD. Doublet identification in single-cell sequencing data using scDblFinder. F1000Res **2021**;10:979
6. Zhang Z, Luo D, Zhong X, Choi JH, Ma Y, Wang S*, et al.* SCINA: A Semi-Supervised Subtyping Algorithm of Single Cells and Bulk Samples. Genes (Basel) **2019**;10(7)
7. Alshetaiwi H, Pervolarakis N, McIntyre LL, Ma D, Nguyen Q, Rath JA*, et al.* Defining the emergence of myeloid-derived suppressor cells in breast cancer using single-cell transcriptomics. Sci Immunol **2020**;5(44)
8. Zhao J, Jaffe A, Li H, Lindenbaum O, Sefik E, Jackson R*, et al.* Detection of differentially abundant cell subpopulations in scRNA-seq data. Proc Natl Acad Sci U S A **2021**;118(22)
9. Lu H, Ping J, Zhou G, Zhao Z, Gao W, Jiang Y*, et al.* CommPath: An R package for inference and analysis of pathway-mediated cell-cell communication chain from single-cell transcriptomics. Comput Struct Biotechnol J **2022**;20:5978-83
10. Tang C, Li C, Tang C, Zhan W, Zheng H, Peng XJAM. Quantitative determination of platinum derived from cisplatin in human plasma ultrafiltrate using derivatization with diethyldithiocarbamate and liquid chromatography coupled with electrospray ionization tandem mass spectrometry. **2013**;5(24):7117-26
11. Christopoulos P, Harel M, McGregor K, Brody Y, Puzanov I, Bar J*, et al.* Plasma Proteome-Based Test for First-Line Treatment Selection in Metastatic Non-Small Cell Lung Cancer. JCO Precis Oncol **2024**;8:e2300555
