# Supplementary material for: PD-1 Blockade–Induced DKK1 Expression by CD8+ T Cells Promotes Blood–Brain Barrier Permeabilization
Source: Cancer Discov. 2026 Jan 13;16(5):976–92. doi: 10.1158/2159-8290.CD-25-1222 (PMC13133603; doi:10.1158/2159-8290.CD-25-1222)
Supplement: Supplementary Table 3 — Log2-fold change in the levels of circulating plasma proteins in BALB/c mice treated with anti-PD1. [file cd-25-1222_supplementary_table_3_suppst3.pdf]

Table S3. Log_2-fold change in the levels of circulating plasma proteins in BALB/c mice treated with anti-PD1.

No.	Candidate protein	Log_2-Fold change (Anti-PD1 to IgG ratio)
1	IL-12 p40	4.55
2	Cystatin C	4.40
3	IL-6	3.96
4	Fetuin A/AHSG	3.86
5	CCL5/RANTES	3.70
6	LDL R	3.02
7	C14	3.00
8	IL-10	2.86
9	M-CSF	2.78
10	Myeloperoxidase	2.74
11	Proliferin	2.54
12	Pref-1/DLK-1/FA1	2.54
13	IL-2	2.46
14	MMP-9	2.43
15	IL-7	2.38
16	DKK-1	2.37
17	CCL17/TARC	2.32
18	Lipocalin-2/NGAL	2.25
19	RBP4	2.18
20	TNF-α	2.12
21	PDGF-BB	2.05
22	HGF	1.96
23	Serpin E1/PAI-1	1.92
24	Chemerin	1.86
25	CD40/TNFRSF5	1.74
26	Pentraxin 3/TSG-14	1.72
27	IL-1rα/IL-1F3	1.61
28	IL-33	1.59
29	WISP-1/CCN4	1.57
30	G-CSF	1.56
31	Endoglin/CD105	1.50
32	Pentraxin 2/SAP	1.44
33	Chitinase 3-like 1	1.41
34	FGF-21	1.31
35	IL-13	1.30
36	IL-22	1.28
37	IL-27 p28	1.20
38	GM-CSF	1.11
39	Gas 6	1.05

40	Periostin/TSG-14	1.02
41	Leptin	1.02
42	IL-28A/B	1.01
43	IFN-γ	0.90
44	Osteprotegerin/TNFRS11B	0.85
45	LIF	0.85
46	CXCL13/BCL/BCA-1	0.84
47	CCL3/CCL4/MIP-1α/β	0.82
48	FGF acidic	0.79
49	CXCL9/MIG	0.79
50	TIM-1/KIM-1/HAVCR	0.78
51	CCL21/6Ckine	0.73
52	Serpin F1/PAI-1	0.71
53	VCAM-1/CD106	0.70
54	E-Selectin/CD62E	0.69
55	IL-4	0.55
56	CCL19/MIP-3β	0.55
57	CCL2/MCP-1	0.52
58	CCL11/Eotaxin	0.52
59	IL-1α/IL-1F1	0.51
60	angiopoietin-1	0.46
61	EGF	0.46
62	CXCL10/IP-10	0.41
63	P-Selectin/CD62P	0.36
64	CCL12/MCP-5	0.32
65	CD160	0.32
66	CCL20/MIP-3α	0.31
67	Proprotein Convertase 9/PCSK9	0.28
68	Thrombopoietin	0.26
69	IL-1β/IL-F2	0.26
70	IGFBP-2	0.24
71	IGFBP-1	0.21
72	LIX	0.15
73	Reg3G	0.10
74	IL-3	0.08
75	IGFP-6	0.07
76	CCL6/C10	0.07
77	MMP-3	0.01
78	ampherigulin	0.00
79	CCL22/MDC	0.00
80	Coagulation factor III/Tissue factor	0.00
81	CXCL1/Fractalkine	0.00
82	CXCL11/I-TAC	0.00

83	CXCL16	0.00
84	CXCL2/MIP-2	0.00
85	IL-23	0.00
86	IL-5	0.00
87	PD-ECGF	0.00
88	IL-17A	-0.05
89	Flt-3 ligand	-0.06
90	ICAM-1/CD54	-0.07
91	angiopoietin-2	-0.10
92	CXCL1/KC	-0.11
93	CRP	-0.14
94	Osteopontin (OPN)	-0.15
95	MMP-2	-0.15
96	BAFF/BlyS/TNSF13B	-0.15
97	Endostaim	-0.16
98	DPPIV/CD26	-0.17
99	Complement component C5/C5α	-0.17
100	GDF-15	-0.27
101	adiponectin	-0.30
102	Resistin	-0.30
103	CD93	-0.38
104	IGFP-5	-0.41
105	Complement Factor D	-0.41
106	angiopoietin-like 3	-0.43
107	RAGE	-0.43
108	IL-11	-0.49
109	IGFBP-3	-0.56
110	IL-15	-0.68
111	VEGF	-1.55

Table S3. Log_2-fold change in the levels of circulating plasma proteins in BALB/c mice treated with anti-PD1. Plasma from non-tumor bearing 8-week-old BALB/c mice treated with anti-PD1 or IgG control for one week was collected, and applied to a protein array kit. Densitometry analysis of the array was performed, and the log2 fold change between anti-PD1 and IgG was calculated.
