# Supplementary material for: PD-1 Blockade–Induced DKK1 Expression by CD8+ T Cells Promotes Blood–Brain Barrier Permeabilization
Source: Cancer Discov. 2026 Jan 13;16(5):976–92. doi: 10.1158/2159-8290.CD-25-1222 (PMC13133603; doi:10.1158/2159-8290.CD-25-1222)
Supplement: Supplementary Table 4 — Clinicopathological features of patients of retrospective study [file cd-25-1222_supplementary_table_4_suppst4.pdf]

Table S4. Clinicopathological features of patients of retrospective study.

No.	Pathology	ICI used for treatment	ICI target	No. of moths between pre and post imaging	Change in intensity (Δ Confluence sinus)
1	NSCLC	Pembrolizumab	Anti-PD1	3	12
2	NSCLC	Pembrolizumab	Anti-PD1	4	-6
3	NSCLC	Pembrolizumab	Anti-PD1	4	33
4	NSCLC	Nivolumab	Anti-PD1	4	-44
5	NSCLC	Pembrolizumab	Anti-PD1	5	22
6	NSCLC	Pembrolizumab	Anti-PD1	5	68
7	NSCLC	Nivolumab	Anti-PD1	5	108
8	NSCLC	Pembrolizumab	Anti-PD1	5	38
9	NSCLC	Pembrolizumab	Anti-PD1	5	-45
10	NSCLC	Nivolumab	Anti-PD1	5	5
11	NSCLC	Pembrolizumab	Anti-PD1	6	-46
12	NSCLC	Pembrolizumab	Anti-PD1	7	6
13	NSCLC	Nivolumab	Anti-PD1	7	133
14	NSCLC	Pembrolizumab	Anti-PD1	8	4
15	NSCLC	Pembrolizumab	Anti-PD1	8	52
16	NSCLC	Pembrolizumab	Anti-PD1	8	-62
17	NSCLC	Pembrolizumab	Anti-PD1	8	-45
18	NSCLC	Nivolumab	Anti-PD1	8	-26
19	NSCLC	Pembrolizumab	Anti-PD1	9	1
20	NSCLC	Pembrolizumab	Anti-PD1	10	39
21	NSCLC	Pembrolizumab	Anti-PD1	11	-72
22	NSCLC	Pembrolizumab	Anti-PD1	12	63

Table S4. Clinicopathological features of patients of retrospective study. Patients with non–small cell lung cancer (NSCLC) underwent T1-weighted MRI before treatment initiation and again 3-12 months after the first cycle of immune checkpoint inhibitor (ICI) therapy. Changes in signal intensity between baseline and follow-up scans were quantified at the confluence of sinuses.
