# Supplementary material for: PD-1 Blockade–Induced DKK1 Expression by CD8+ T Cells Promotes Blood–Brain Barrier Permeabilization
Source: Cancer Discov. 2026 Jan 13;16(5):976–92. doi: 10.1158/2159-8290.CD-25-1222 (PMC13133603; doi:10.1158/2159-8290.CD-25-1222)
Supplement: Supplementary Table 5 — Clinicopathological features of patients of prospective study [file cd-25-1222_supplementary_table_5_suppst5.pdf]

Table S5. Clinicopathological features of patients of prospective study

No.	Gender	Pathology	ICI	ICI target	No. of cycles between imaging	Radiological findings
1	Male	NSCLC	Durvalumab	Anti-PDL1	4	None
2	Female	NSCLC	Pembrolizumab	Anti-PD1	5	Occipitoparietal enhancement
3	Male	NSCLC	Pembrolizumab	Anti-PD1	4	Left frontal enhancement. Left occipital enhancement
4	Female	SCLC	Pembrolizumab	Anti-PD1	5	Right parietooccipital enhancement. Left midline occipital cortical enhancement
5	Male	NSCLC	Durvalumab	Anti-PDL1	5	None

Table S5. Clinicopathological features of patients of prospective study. Patients with non-small cell lung cancer (NSCLC) or small cell lung cancer (SCLC) were treated with anti-PD1 or anti-PDL1 for 4-5 cycles. 3D FLAIR MRI images were taken. ICI, immune checkpoint blockade.
