# Supplementary material for: PD-1 Blockade–Induced DKK1 Expression by CD8+ T Cells Promotes Blood–Brain Barrier Permeabilization
Source: Cancer Discov. 2026 Jan 13;16(5):976–92. doi: 10.1158/2159-8290.CD-25-1222 (PMC13133603; doi:10.1158/2159-8290.CD-25-1222)
Supplement: Supplementary Table 6 — Association of longitudinal increase in plasma DKK1 levels with brain metastasis [file cd-25-1222_supplementary_table_6_suppst6.pdf]

Table S6. Association of longitudinal increase in plasma DKK1 levels with brain metastasis in NSCLC patients undergoing anti-PD1 treatment.

Progression-free survival event	Plasma DKK1 increase following anti-PD1 treatment	Incidence of brain metastasis	*p*-value
No progression (102)	≤ 1.5-fold (87)	26.4% (23)	0.5804
	>1.5-fold (15)	33.3% (5)	
Progression (144)	≤ 1.5-fold (121)	23.1% (28)	0.0425
	>1.5-fold (23)	43.4% (10)	

Overall survival event	Plasma DKK1 increase following anti-PD1 treatment	Incidence of brain metastasis	*p*-value
Alive (129)	≤ 1.5-fold (108)	25.0% (27)	0.4277
	>1.5-fold (21)	33.3% (7)	
Dead (117)	≤ 1.5-fold (100)	24.0% (24)	0.0486
	>1.5-fold (17)	47.0% (8)	

Table S6. Association of longitudinal increase in plasma DKK1 levels with brain metastasis in NSCLC patients undergoing anti-PD1 treatment. A published dataset of plasma profiles from NSCLC patients collected before and after initial immunotherapy was used (11). Only patients receiving anti-PD1 monotherapy or anti-PD1+anti-CTLA-4 (n=246) were included; PD-L1–treated patients were excluded due to low numbers (n=8). Patients were stratified by post-treatment DKK1 fold-change, defining significant upregulation as a >1.5-fold increase. Numbers in parenthesis indicate absolute number of patients.
