# Supplementary material for: PD-1 Blockade–Induced DKK1 Expression by CD8+ T Cells Promotes Blood–Brain Barrier Permeabilization
Source: Cancer Discov. 2026 Jan 13;16(5):976–92. doi: 10.1158/2159-8290.CD-25-1222 (PMC13133603; doi:10.1158/2159-8290.CD-25-1222)
Supplement: Supplementary Table 7 — Cell surface markers analyzed using flow cytometry. [file cd-25-1222_supplementary_table_7_suppst7.pdf]

Table S7. Cell surface markers analyzed using flow cytometry.

**Cell types**	**Surface markers**
B cells	$CD45^{+}B220^{+}$
CD4+ T cells	$CD45^{+}CD4^{+}$
CD8+ T cells	$CD45^{+}CD8^{+}$
Activated CD8+ T cells	$CD45^{+}CD8^{+}CD25^{+}$
Natural killer cells	CD45+CD49b+ or $CD45^{+}NKp46^{+}$
Dendritic cells	$CD45^{+}CD11c^{+}$
M-MDSCs	$CD45^{+}CD11b^{+}Ly6C^{+}Ly6G^{low}$
G-MDSCs	$CD45^{+}CD11b^{+}Ly6C^{low}Ly6G^{+}$
Macrophages	$CD45^{+}CD11b^{+}F4/80^{+}$

Table S7. Cell surface markers analyzed using flow cytometry. Cells were incubated with antibody mixture, and subsequently analyzed by flow cytometry. The cells were annotated based on the different antibody composition indicated in the table.
