# Supplementary material for: PD-1 Blockade–Induced DKK1 Expression by CD8+ T Cells Promotes Blood–Brain Barrier Permeabilization
Source: Cancer Discov. 2026 Jan 13;16(5):976–92. doi: 10.1158/2159-8290.CD-25-1222 (PMC13133603; doi:10.1158/2159-8290.CD-25-1222)
Supplement: Supplementary Table 8 — List of primers sequences used for qPCR analysis of specific genes [file cd-25-1222_supplementary_table_8_suppst8.pdf]

Table S8. List of primers sequences used for qPCR analysis of specific genes.

Gene	Forward primer	Revere primer
CD25	5-'CACTACGAGTGTATTCCGGGA-3'	5-'TCGGTGGTGTTCTCTTTCATCT-3'
Dkk1	5-'GTAATGACCACAACGCCGC-3'	5-'TCTGACCACAGCCATTTTCCT-3'
Ctnnb1	5-'ATCTTAAGCCCTCGCTCGGT-3'	5-'CAGGTCAGCTTGAGTAGCCA-3'
Tcf7	5-'AGGTGGCATGCACTATCTCG-3'	5-'TGCATTTCTTTTTCCTCCTGTGG-3'
Foxm1	5-'GGAGGAAATGCCACACTTAGCG-3'	5-'TAGGACTTCTTGGGTCTTGGGGTG-3'
Gapdh	5-'TGACCACAGTCCATGCCATC-3'	5-'GACGGACACATTGGGGGTAG-3'

Table S8. List of primers sequences used for qPCR analysis of specific genes. CD8+ T cells isolated from the spleen of mice, and underwent various procedures as indicated in the text, were prepared for RNA extraction. The sequence of the various primers for each gene expression is provided in the table.
