# Supplementary material for: PD-1 Blockade–Induced DKK1 Expression by CD8+ T Cells Promotes Blood–Brain Barrier Permeabilization
Source: Cancer Discov. 2026 Jan 13;16(5):976–92. doi: 10.1158/2159-8290.CD-25-1222 (PMC13133603; doi:10.1158/2159-8290.CD-25-1222)
Supplement: Supplementary Figure 1 — Dot plot of cell populations identified in scRNA-seq data [file cd-25-1222_supplementary_figure_1_suppsf1.pdf]

FIGURE S1

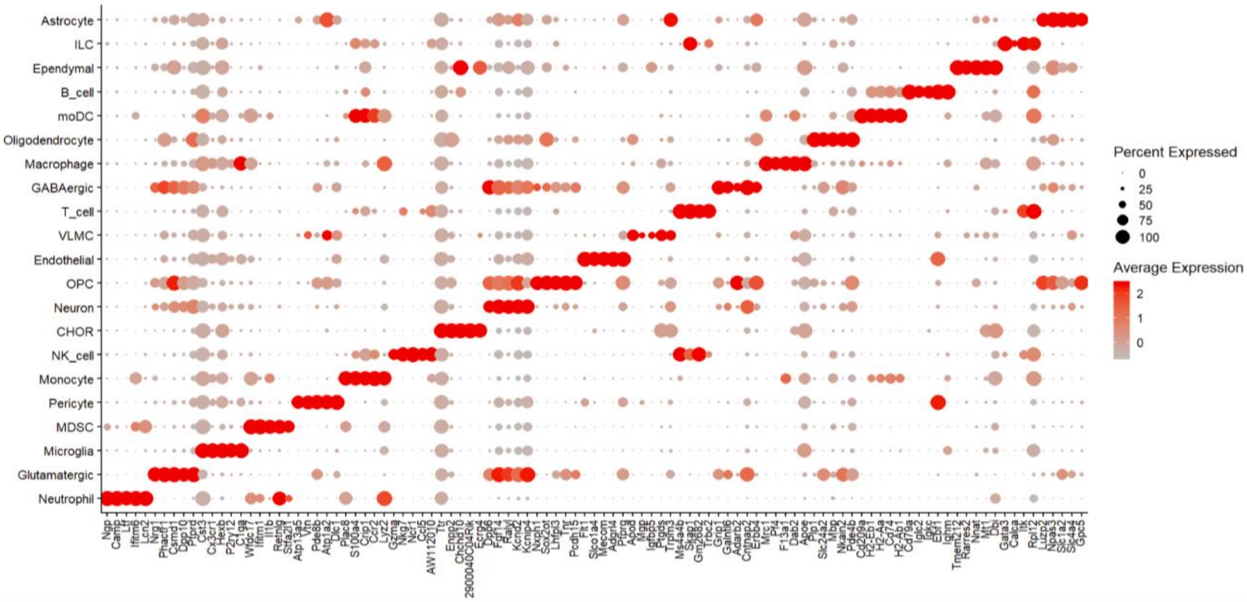

Fig. S1. Dot plot of cell populations identified in scRNA-seq data. Supervised clustering revealed 21 populations in scRNA-seq dataset. The figure shows the top-5 differentially expressed genes in individual populations. The size of each dot indicates the percentage of expression, while the intensity of color from grey to red reflects the range from low to high average gene expression.
