# Supplementary material for: PD-1 Blockade–Induced DKK1 Expression by CD8+ T Cells Promotes Blood–Brain Barrier Permeabilization
Source: Cancer Discov. 2026 Jan 13;16(5):976–92. doi: 10.1158/2159-8290.CD-25-1222 (PMC13133603; doi:10.1158/2159-8290.CD-25-1222)
Supplement: Supplementary Figure 2 — Gating strategy for flow cytometry analysis [file cd-25-1222_supplementary_figure_2_suppsf2.pdf]

FIGURE S2

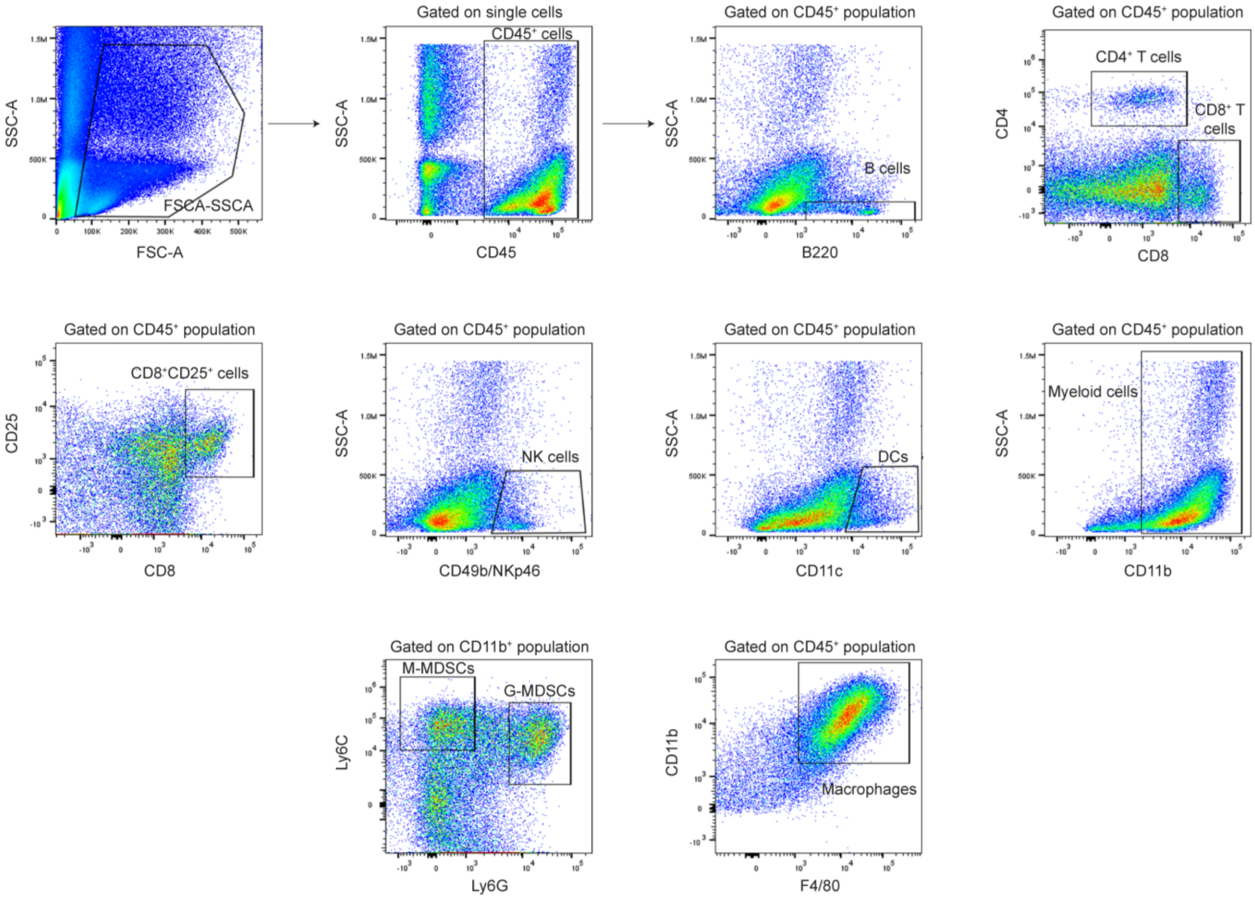

Fig. S2. Gating strategy for flow cytometry analysis. Representative flow cytometry plots showing the gating strategy used to identify various immune cell populations including B cells, CD4^{+} T cells, CD8^{+} T cells, activated CD8^{+} T cells (CD8^{+}CD25^{+}), natural killer (NK) cells, dendritic cells (DCs), monocytic and granulocytic myeloid-derived suppressor cells (M-MDSCs and G-MDSCs), and macrophages, from the tumor-free brains of BALB/c mice bearing orthotopic EMT6 breast tumors and treated with either IgG or anti-PD1, used to validate the scRNA-seq findings.
