# Supplementary material for: PD-1 Blockade–Induced DKK1 Expression by CD8+ T Cells Promotes Blood–Brain Barrier Permeabilization
Source: Cancer Discov. 2026 Jan 13;16(5):976–92. doi: 10.1158/2159-8290.CD-25-1222 (PMC13133603; doi:10.1158/2159-8290.CD-25-1222)
Supplement: Supplementary Figure 3 — Validation of scRNA-seq data using flow cytometry [file cd-25-1222_supplementary_figure_3_suppsf3.pdf]

FIGURE S3

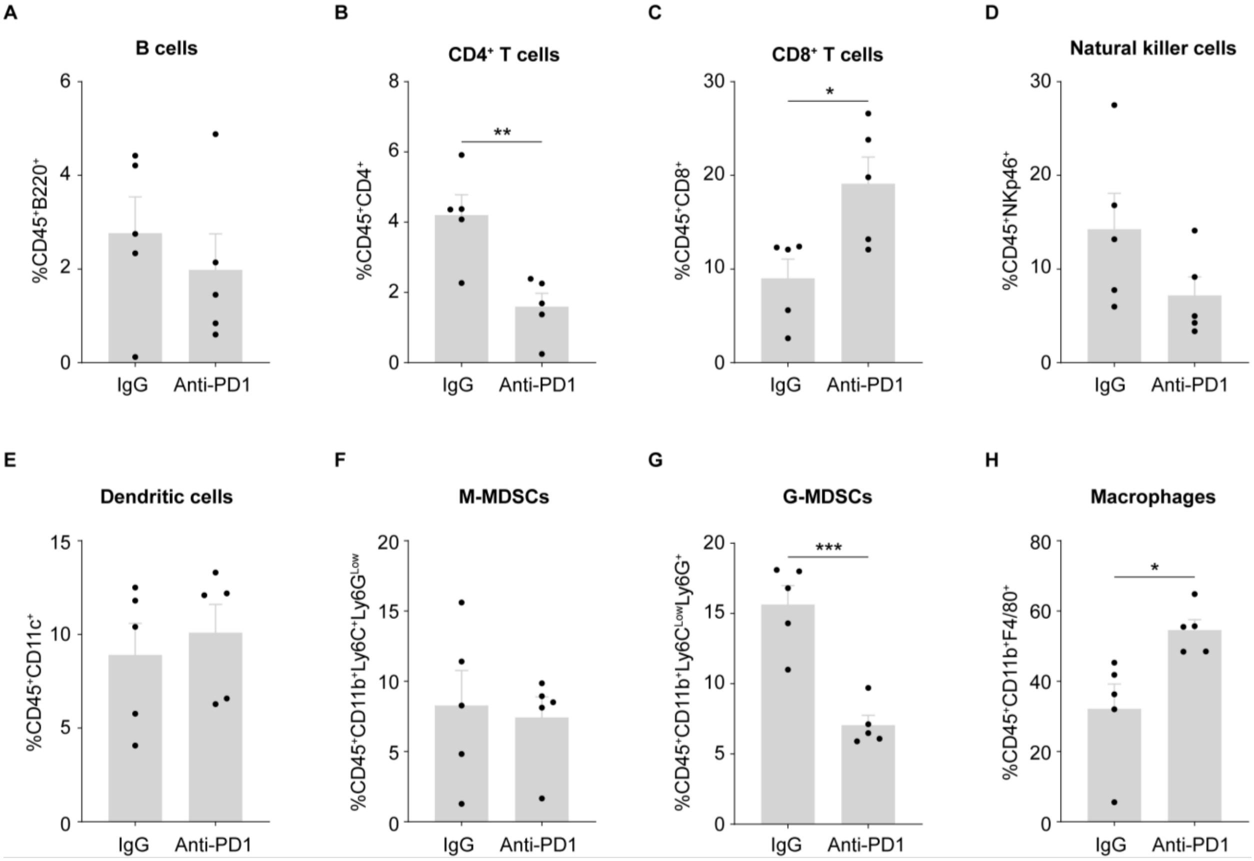

Fig. S3. Validation of scRNA-seq data using flow cytometry. Flow cytometry was employed to validate the relative abundance of various immune cell populations identified in the scRNA-seq data (Figure 1). Bar plots showing percentages of different immune cell populations, including **(A)** B cells, **(B)** $CD4^+$ T cells, **(C)** $CD8^+$ T cells, **(D)** natural killer cells, **(E)** dendritic cells, **(F)** monocytic- and **(G)** granulocytic-myeloid derived suppressor cells (M- and G-MDSCs), and **(H)** macrophages in the tumor-free brains of IgG- and anti-PD1-treated 8-week-old BALB/c mice bearing orthotopic EMT6 breast cancer cells (n=5 mice/group). Significance was assessed by means of Student's t-test (*p<0.05, ***p*<0.01, ****p*<0.001).
