# Supplementary material for: PD-1 Blockade–Induced DKK1 Expression by CD8+ T Cells Promotes Blood–Brain Barrier Permeabilization
Source: Cancer Discov. 2026 Jan 13;16(5):976–92. doi: 10.1158/2159-8290.CD-25-1222 (PMC13133603; doi:10.1158/2159-8290.CD-25-1222)
Supplement: Supplementary Figure 4 — Anti-PD1 therapy induces genetic alterations in myeloid-derived suppressor cells within the brain [file cd-25-1222_supplementary_figure_4_suppsf4.pdf]

FIGURE S4

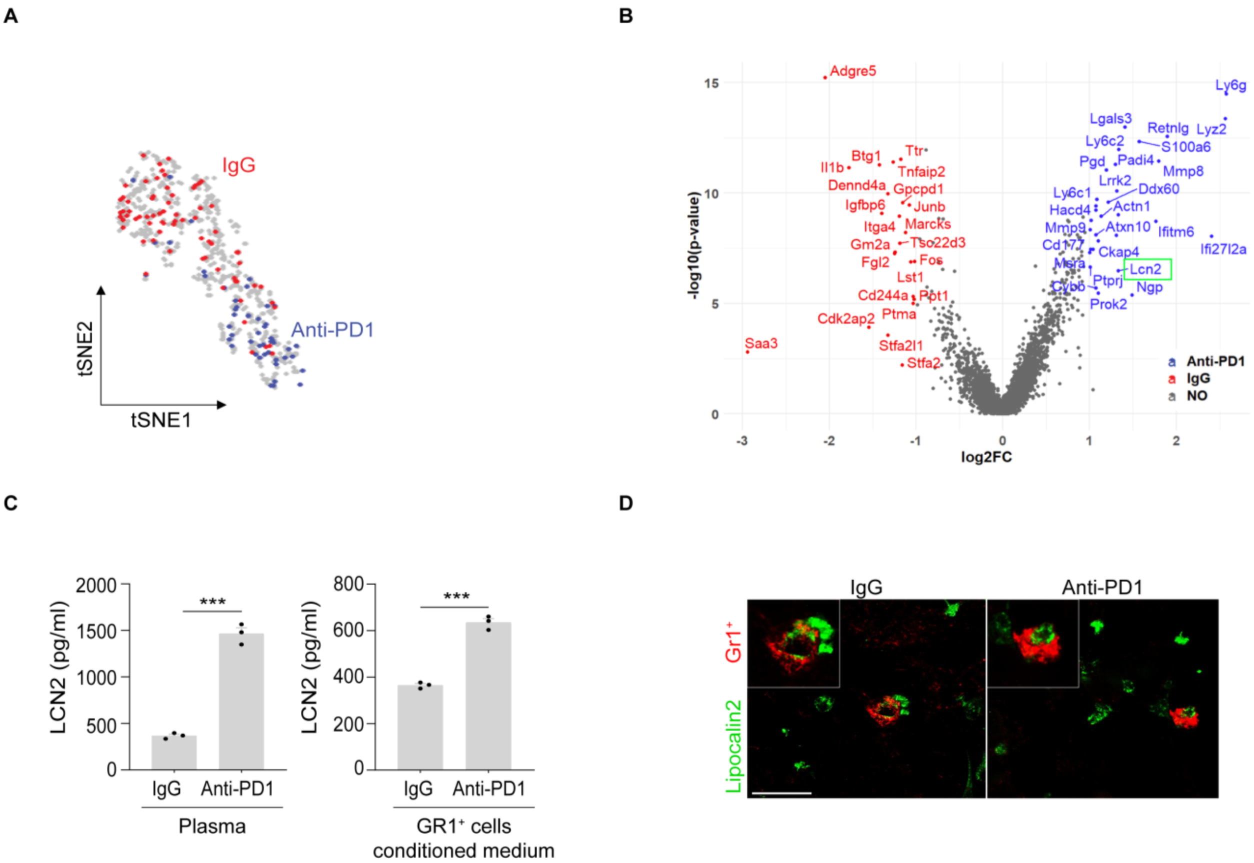

Fig. S4. Anti-PD1 therapy induces genetic alterations in myeloid-derived suppressor cells within the brain. Eight-week-old BALB/c mice bearing orthotopic EMT6 breast tumors received treatment with either anti-PD1 antibody or IgG control. Following one week of treatment, the mice were perfused, and their brains were collected, dissociated into single-cell suspensions, and processed for single-cell RNA sequencing (scRNA-seq). **(A)** A tSNE plot displaying two subpopulations of myeloid derived suppressor cells (MDSCs) in the brains of IgG and anti-PD1 treated mice, based on differential abundance (DA). **(B)** A volcano plot illustrating fold changes of differentially expressed genes (DEGs) in subpopulations of MDSCs, marked by red dots (upregulation in IgG), blue dots (upregulation in anti-PD1), and gray dots (no change). Green box highlights Lipocalin-2 (LCN2). **(C)** LCN2 levels were analyzed using specific ELISA in the plasma and conditioned media of $Gr1^+$ cells harvested from the spleens of IgG- and anti-PD1 treated 8-week-old BALB/c mice bearing orthotopic breast tumors of EMT6 cells. Bar graphs are plotted. **(D)** Representative immunofluorescence images showing LCN2 (green) and $Gr1^+$ cells (red) in the brains of IgG- and anti-PD1 treated mice. White boxes (top left) indicate magnified areas (40X magnification and 1.3X digital zoom). Scale bar: 20 μm. Significance was assessed by Student's t-test for (C) (***p<0.001).
