# Supplementary material for: PD-1 Blockade–Induced DKK1 Expression by CD8+ T Cells Promotes Blood–Brain Barrier Permeabilization
Source: Cancer Discov. 2026 Jan 13;16(5):976–92. doi: 10.1158/2159-8290.CD-25-1222 (PMC13133603; doi:10.1158/2159-8290.CD-25-1222)
Supplement: Supplementary Figure 5 — DCE-MRI assessment demonstrates alterations in the blood-brain barrier of anti-PD1 treated mice [file cd-25-1222_supplementary_figure_5_suppsf5.pdf]

FIGURE S5

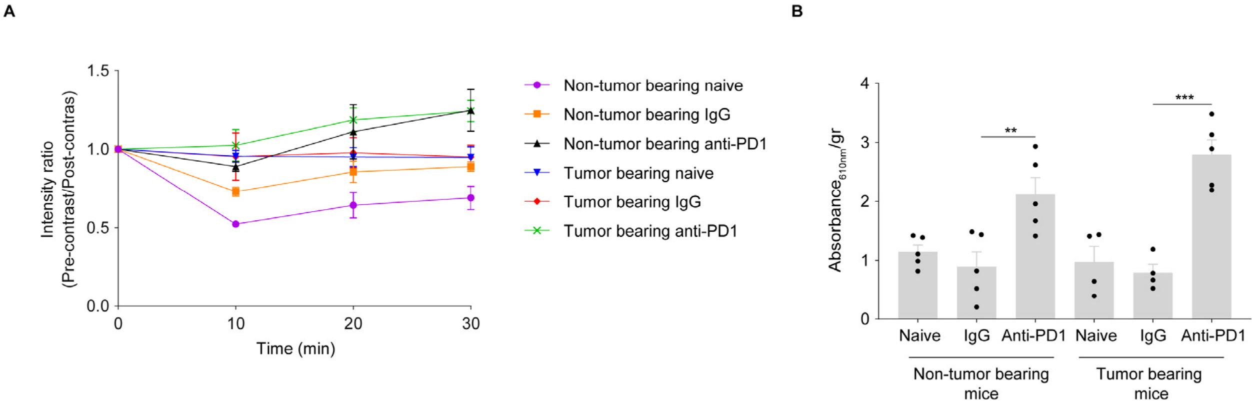

Fig. S5. DCE-MRI assessment demonstrates alterations in the blood-brain barrier of anti-PD1 treated mice. (A) Eight-week-old BALB/c mice, either non–tumor-bearing or bearing orthotopic EMT6 breast tumors, were left untreated or treated with IgG or anti–PD-1. Blood–brain barrier permeability was assessed by DCE-MRI, and the change in gadolinium enhancement was quantified as the ratio of pre-contrast (t=0) to post-contrast signal intensity over 30 minutes (n=3–4 mice/group). **(B)** Evans Blue extravasation in the brain was assessed in an independent cohort of mice under a similar experimental setting, and quantification is presented as bar graphs (n=4-5 mice/group). Significance was assessed using one-way ANOVA (**p<0.01, and ***p<0.001).
