# Supplementary material for: PD-1 Blockade–Induced DKK1 Expression by CD8+ T Cells Promotes Blood–Brain Barrier Permeabilization
Source: Cancer Discov. 2026 Jan 13;16(5):976–92. doi: 10.1158/2159-8290.CD-25-1222 (PMC13133603; doi:10.1158/2159-8290.CD-25-1222)
Supplement: Supplementary Figure 6 — Assessment of BBB permeability in response to different immune checkpoint inhibitors [file cd-25-1222_supplementary_figure_6_suppsf6.pdf]

FIGURE S6

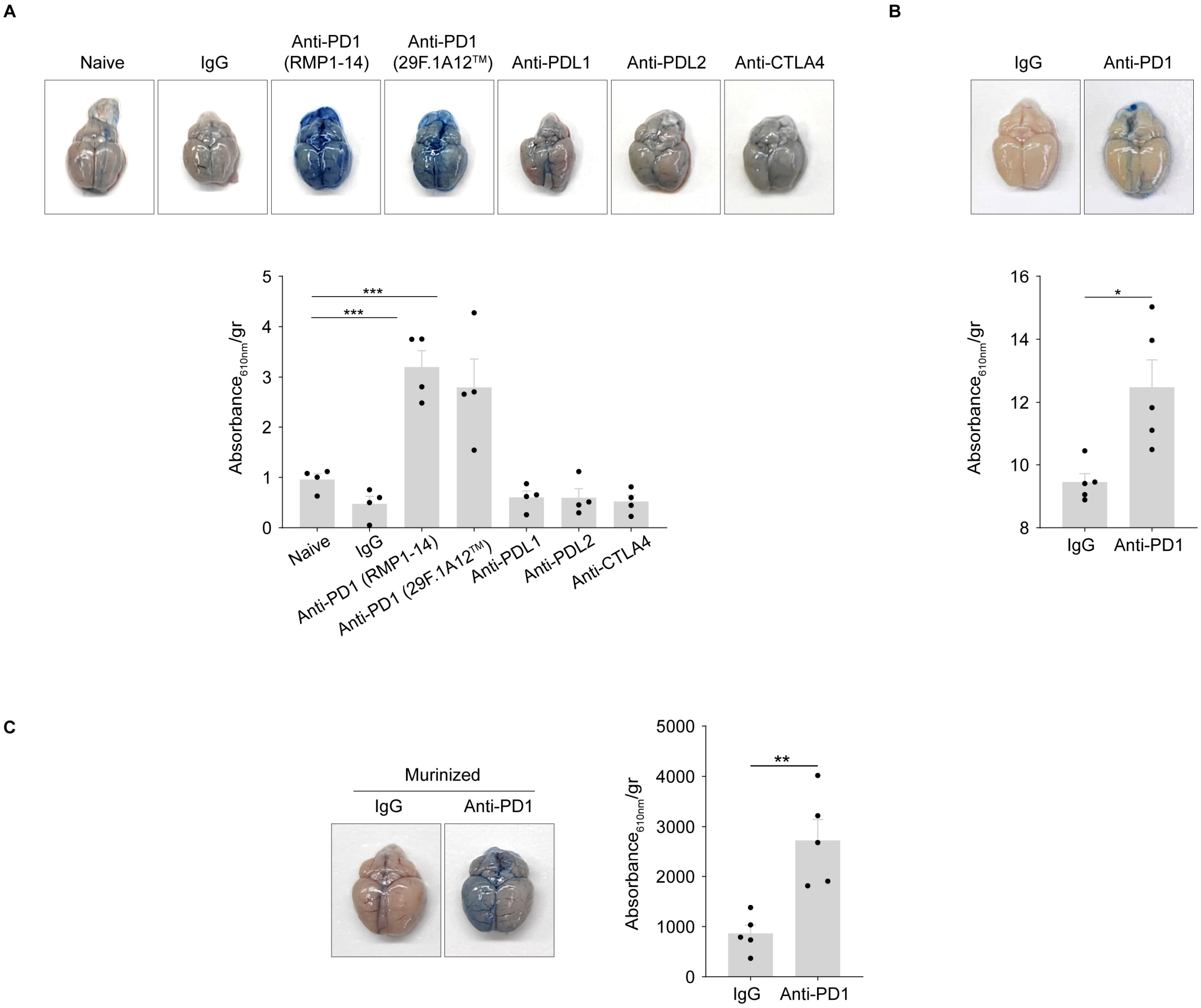

Fig. S6. Assessment of BBB permeability in response to different immune checkpoint inhibitors therapies using Evans Blue dye delivery methods. Representative images of Evans Blue (EB)-perfused brains from 8-week-old BALB/c mice illustrating: **(A)** the effect of various anti-rat immune checkpoint inhibitors (ICIs), including naive (untreated), IgG control, anti-PD1 (clones RMP1-14 and 29F.1A12™), anti-PDL1, anti-PDL2, and anti-CTLA4 (n=4 mice/group); **(B)** the effect of EB dye administration route, comparing anti-rat IgG and anti-PD1–treated mice in which the dye was administered via the tail vein; and **(C)** the effect of anti-mouse (murinized) IgG and anti-PD1 antibodies. All images are followed by absorbance quantification. The absorbance of EB dye extracted per gram of brain tissue is plotted. Statistical analysis was performed using one-way ANOVA for (A) and Student's t-test for (B-C) (*$p < 0.05$, **$p < 0.01$, ***$p < 0.001$).
