# Supplementary material for: PD-1 Blockade–Induced DKK1 Expression by CD8+ T Cells Promotes Blood–Brain Barrier Permeabilization
Source: Cancer Discov. 2026 Jan 13;16(5):976–92. doi: 10.1158/2159-8290.CD-25-1222 (PMC13133603; doi:10.1158/2159-8290.CD-25-1222)
Supplement: Supplementary Figure 7 — Mice primed with anti-PD1 therapy exhibited increased brain metastasis of lung cancer cells [file cd-25-1222_supplementary_figure_7_suppsf7.pdf]

FIGURE S7

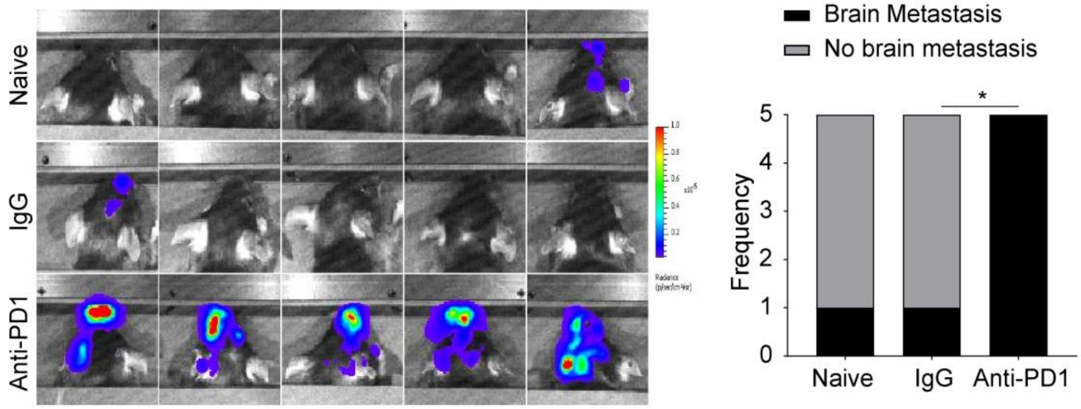

Fig. S7. Mice primed with anti-PD1 therapy exhibited increased brain metastasis of lung cancer cells. Bioluminescence brain images of naive (untreated), IgG- and anti-PD1-treated 8-week-old C57BL/6 mice injected with LLC cells in an experimental brain metastasis assay (n=5 mice/group). The frequency of metastasis is plotted. Significance was assessed using Fisher's exact test (*$p<0.05$).
