# Supplementary material for: PD-1 Blockade–Induced DKK1 Expression by CD8+ T Cells Promotes Blood–Brain Barrier Permeabilization
Source: Cancer Discov. 2026 Jan 13;16(5):976–92. doi: 10.1158/2159-8290.CD-25-1222 (PMC13133603; doi:10.1158/2159-8290.CD-25-1222)
Supplement: Supplementary Figure 8 — DKK1 in plasma from anti-PD1 treated mice disrupts the integrity of endothelial cells [file cd-25-1222_supplementary_figure_8_suppsf8.pdf]

FIGURE S8

A

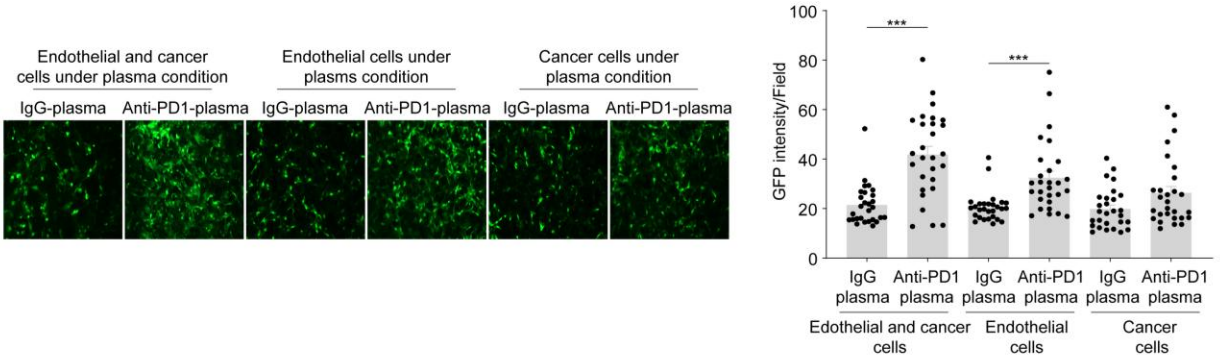

B

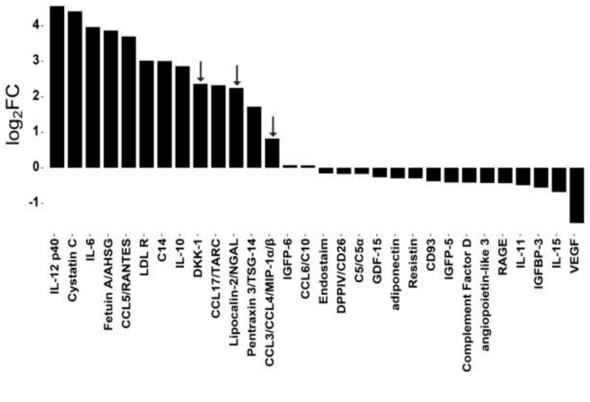

C

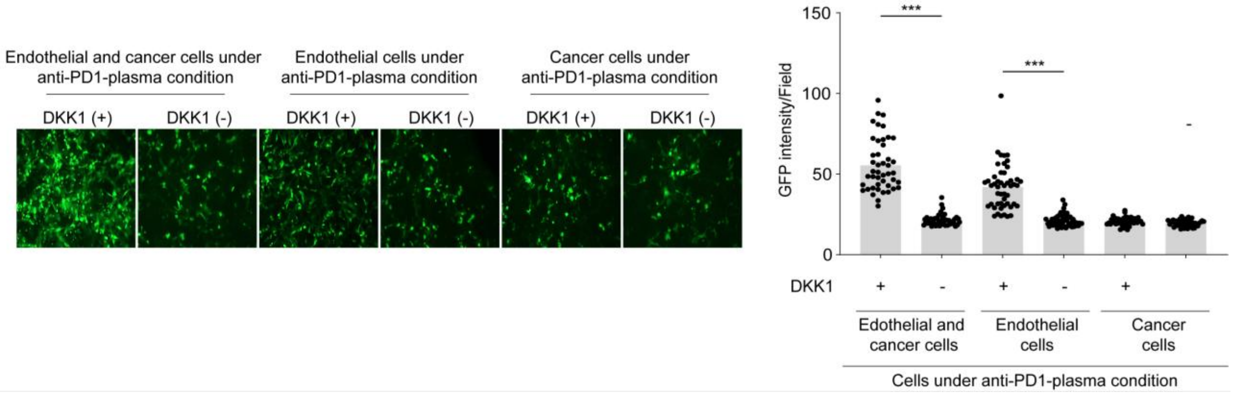

Fig. S8. DKK1 in plasma from anti-PD1 treated mice disrupts the integrity of endothelial cells *in vitro*. (A) Representative images showing migrated fraction of EMT6 breast cancer cells (tagged with GFP) in a trans-endothelial cell migration assay under separate and combined treatments with IgG and anti-PD1 plasma on both endothelial and cancer cells. Migrated fraction of cancer cells is plotted. **(B)** Plasma from BALB/c mice treated with IgG and anti-PD1 were subjected to a cytokine and chemokine protein array. The levels of selected proteins are shown as $\log_2$-fold change (anti-PD1 over IgG). Arrows indicate plasma proteins known to compromise the BBB integrity. **(C)** Representative images displaying the migrated fraction of EMT6 breast cancer cells (tagged with GFP) in a trans-endothelial cell migration assay under various treatments, including separate and combined administration of anti-PD1 plasma with and without DKK1 depletion (+/-) in both endothelial and cancer cells. Migrated fraction of cancer cells is plotted. Significance was assessed by one-way ANOVA (***p<0.001).
