# Supplementary material for: PD-1 Blockade–Induced DKK1 Expression by CD8+ T Cells Promotes Blood–Brain Barrier Permeabilization
Source: Cancer Discov. 2026 Jan 13;16(5):976–92. doi: 10.1158/2159-8290.CD-25-1222 (PMC13133603; doi:10.1158/2159-8290.CD-25-1222)
Supplement: Supplementary Figure 9 — The effect of DKK1 on β-catenin–regulated tight junction proteins in endothelial cell [file cd-25-1222_supplementary_figure_9_suppsf9.pdf]

FIGURE S9

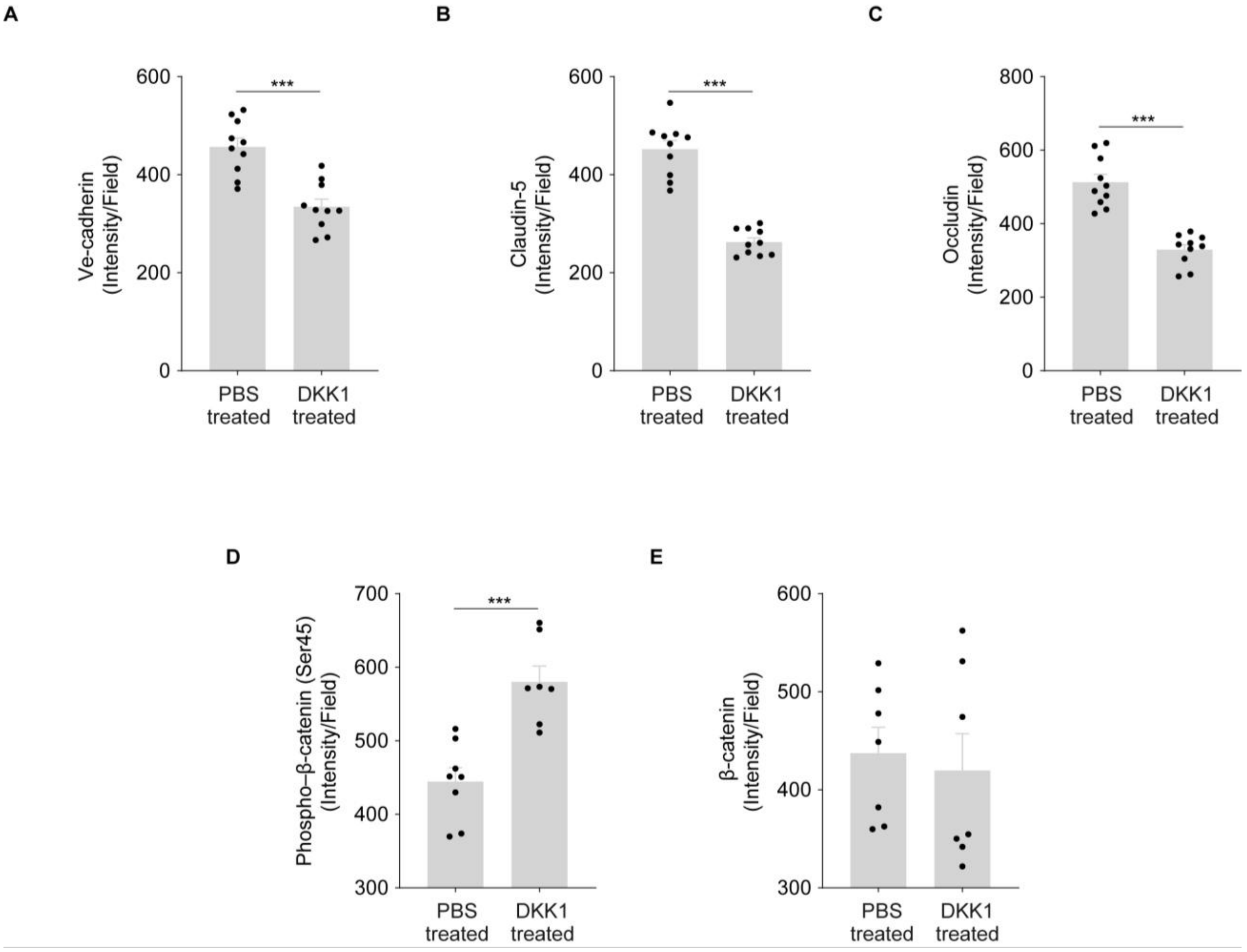

Fig. S9. The effect of DKK1 on β-catenin–regulated tight junction proteins in endothelial cell *in vitro*. bEnd.5 brain endothelial cells were treated with PBS (control) or recombinant DKK1 (100 ng/mL) for 24 hrs, and immunostained for tight and adherens junction proteins, along with markers of β-catenin activation. Bar graphs show the relative fluorescence intensities of **(A)** VE-cadherin, **(B)** Claudin-5, **(C)** Occludin, **(D)** phospho-β-catenin (Ser45), and **(E)** total β-catenin (n=7-10 fields/group). Significance was assessed by Student's t-test (***$p<0.001$).
