# Supplementary material for: PD-1 Blockade–Induced DKK1 Expression by CD8+ T Cells Promotes Blood–Brain Barrier Permeabilization
Source: Cancer Discov. 2026 Jan 13;16(5):976–92. doi: 10.1158/2159-8290.CD-25-1222 (PMC13133603; doi:10.1158/2159-8290.CD-25-1222)
Supplement: Supplementary Figure 10 — Transfusing plasma from anti-PD1-treated BALB/c mice into SCID mice leads to BBB opening [file cd-25-1222_supplementary_figure_10_suppsf10.pdf]

FIGURE S10

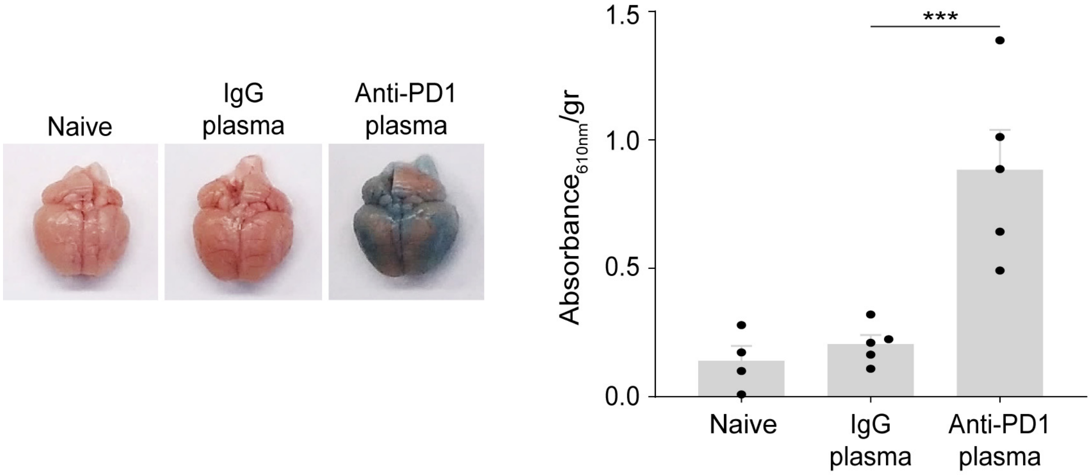

Fig. S10. Transfusing plasma from anti-PD1-treated BALB/c mice into SCID mice leads to BBB opening. Eight-week-old BALB/c mice were treated with IgG, anti-PD1, or left untreated (naive). After one week, plasma was obtained and administered trice in one week to SCID mice (100µl/dose)(n=4-5 mice/group). Representative images of Evans blue (EB) perfused brains of SCID mice are shown, along with graph bars plotting the absorbance of EB dye extracted per gram of brain tissue. Significance was assessed by means of a one-way ANOVA (***p<0.001).
