# Supplementary material for: PD-1 Blockade–Induced DKK1 Expression by CD8+ T Cells Promotes Blood–Brain Barrier Permeabilization
Source: Cancer Discov. 2026 Jan 13;16(5):976–92. doi: 10.1158/2159-8290.CD-25-1222 (PMC13133603; doi:10.1158/2159-8290.CD-25-1222)
Supplement: Supplementary Figure 11 — Dkk1 knockdown in CD8+ T cells [file cd-25-1222_supplementary_figure_11_suppsf11.pdf]

**FIGURE S11**

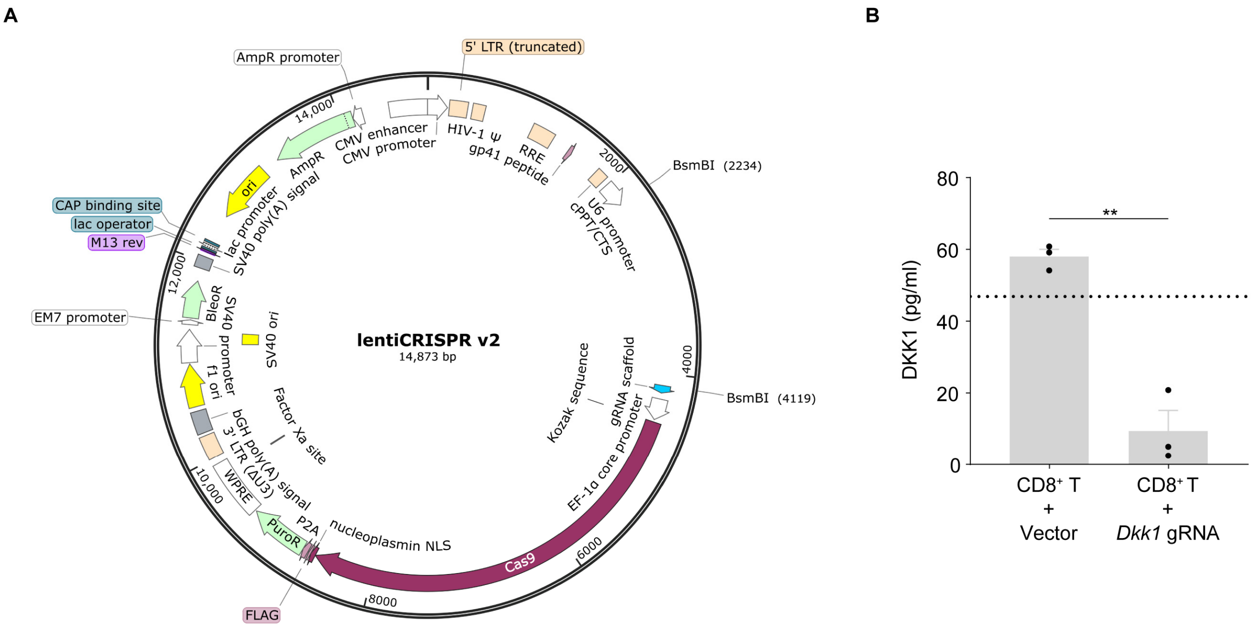


**Fig. S11.** ***Dkk1* knockdown in CD8$^+$ T cells**. **(A)** lentiCRISPR v2 vector map is shown. **(B)** *Dkk1* knockdown was confirmed through ELISA performed on the conditioned media of CD8$^+$ T cells harboring lentiCRISPR v2 vector (control) or the lentiCRISPR v2 vector containing *Dkk1*-specific gRNA. Bar graph showing relative levels of DKK1 in the conditioned media of CD8$^+$ T cells is plotted (Dotted line represents detection limit). Significance was assessed by Student's t-test (***p*<0.01).
