# Supplementary material for: PD-1 Blockade–Induced DKK1 Expression by CD8+ T Cells Promotes Blood–Brain Barrier Permeabilization
Source: Cancer Discov. 2026 Jan 13;16(5):976–92. doi: 10.1158/2159-8290.CD-25-1222 (PMC13133603; doi:10.1158/2159-8290.CD-25-1222)
Supplement: Supplementary Figure 12 — Flow cytometry analysis of PD-L1 and PD-L2 [file cd-25-1222_supplementary_figure_12_suppsf12.pdf]

FIGURE S12

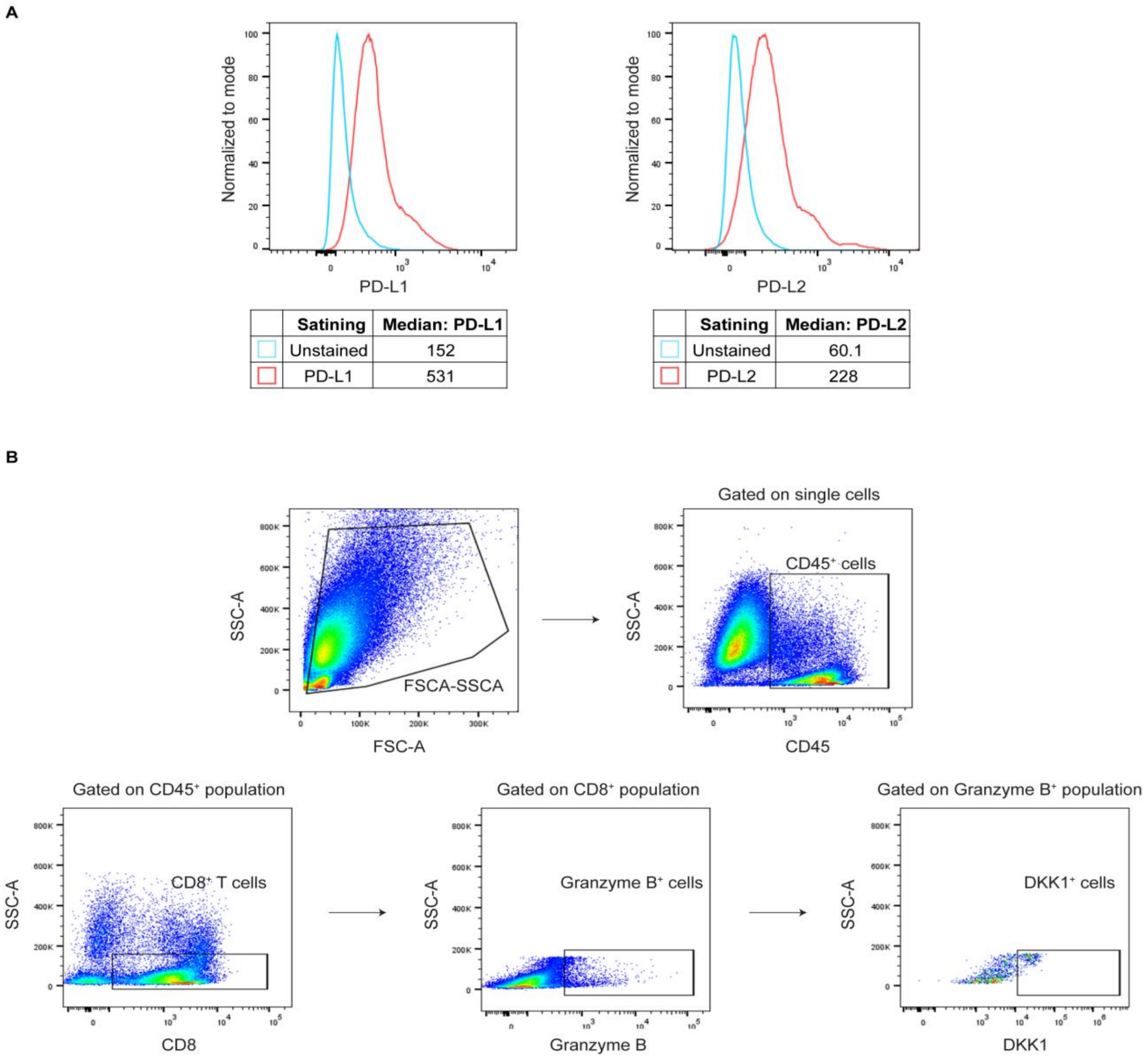

Fig. S12. Flow cytometry analysis of PD-L1 and PD-L2 along with gating strategy for DKK1^{+} activated CD8^{+} T cells. (A) Flow cytometry was used to analyze the expression levels of PD-1 ligands, i.e., PD-L1 and PD-L2, on LLC cells immunostained with specific antibodies. Histograms display the average expression levels of PD-L1 and PD-L2 as well as unstained LLC cells serving as a negative control. The accompanying tables summarize the median expression levels of PD-L1 and PD-L2 in both unstained and stained LLC cell samples. The experiments were performed in triplicate. **(B)** Representative flow cytometry plots showing the gating strategy used to identify and analyses activated CD8^{+} T cells (CD45^{+}CD8^{+}GranzymeB^{+}) and DKK1^{+} activated CD8^{+} T cells (CD45^{+}CD8^{+}GranzymeB^{+}DKK1^{+}) in co-culture of CD8^{+} T cells and LLC cells under various ICI-treated conditions.
