# Supplementary material for: PD-1 Blockade–Induced DKK1 Expression by CD8+ T Cells Promotes Blood–Brain Barrier Permeabilization
Source: Cancer Discov. 2026 Jan 13;16(5):976–92. doi: 10.1158/2159-8290.CD-25-1222 (PMC13133603; doi:10.1158/2159-8290.CD-25-1222)
Supplement: Supplementary Figure 13 — The percentage of DKK1+ on active CD8+ T cells under different ICI-treated conditions [file cd-25-1222_supplementary_figure_13_suppsf13.pdf]

FIGURE S13

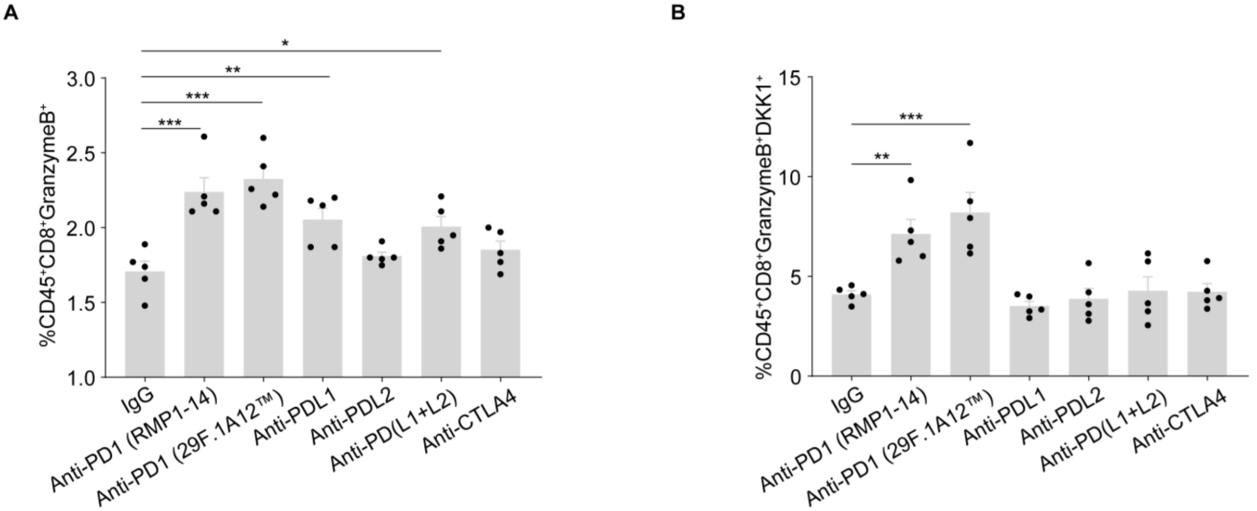

Fig. S13. The percentage of DKK1⁺ on active CD8⁺ T cells under different ICI-treated conditions. CD8⁺ T cells were isolated from the spleens of 8-week-old C57BL/6 mice, activated with PMA (10 ng/ml) and Ionomycin (10 µg/ml), and co-cultured with LLC cancer cells under various ICI treated conditions, as shown in the figure. Flow cytometry was employed to analyze the relative abundance of **(A)** activated CD8⁺ T cells (CD45⁺CD8⁺GranzymeB⁺) and **(B)** DKK1⁺ activated CD8⁺ T cells (CD45⁺CD8⁺GranzymeB⁺DKK1⁺) under various ICI treated conditions (n=5 biological repeats). A bar graph is plotted. Significance was assessed by one-way ANOVA (*p<0.05, **p<0.01, ***p<0.05).
