# Supplementary material for: PD-1 Blockade–Induced DKK1 Expression by CD8+ T Cells Promotes Blood–Brain Barrier Permeabilization
Source: Cancer Discov. 2026 Jan 13;16(5):976–92. doi: 10.1158/2159-8290.CD-25-1222 (PMC13133603; doi:10.1158/2159-8290.CD-25-1222)
Supplement: Supplementary Figure 14 — Effect of Ctnnb1 knockdown on Dkk1 expression in CD8⁺ T cells [file cd-25-1222_supplementary_figure_14_suppsf14.pdf]

FIGURE S14

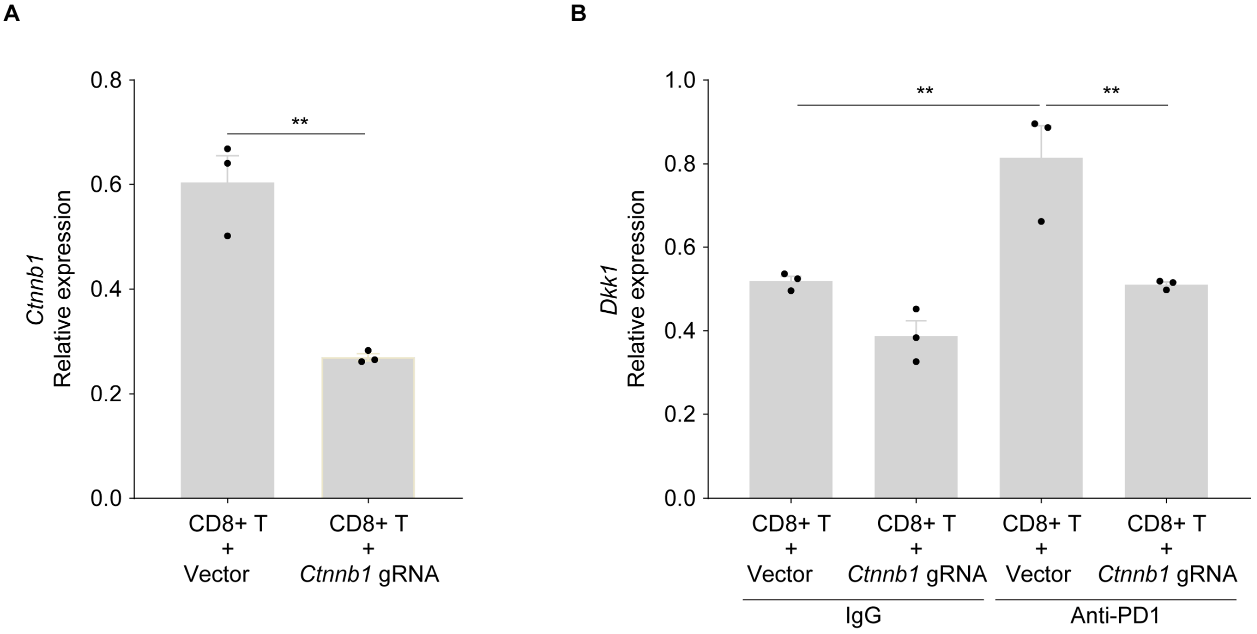

Fig. S14. Effect of *Ctnnb1* knockdown on *Dkk1* expression in CD8⁺ T cells. **(A)** Bar graph showing relative *Ctnnb1* transcript levels in control and *Ctnnb1*-knockdown [guide RNA (gRNA)] $CD8^+$ T cells is plotted. **(B)** Bar graph showing relative *Dkk1* transcript levels in control and *Ctnnb1*-knockdown $CD8^+$ T cells under IgG- and anti-PD1-treated conditions is plotted. Significance was assessed by Student's t-test for (A) and one-way ANOVA for (B) ($**p<0.01$).
