# Supplementary material for: PD-1 Blockade–Induced DKK1 Expression by CD8+ T Cells Promotes Blood–Brain Barrier Permeabilization
Source: Cancer Discov. 2026 Jan 13;16(5):976–92. doi: 10.1158/2159-8290.CD-25-1222 (PMC13133603; doi:10.1158/2159-8290.CD-25-1222)
Supplement: Supplementary Figure 15 — Effect of anti-PD1 therapy on the intracranial progression of sensitive vs. resistant lung cancer cells. [file cd-25-1222_supplementary_figure_15_suppsf15.pdf]

FIGURE S15

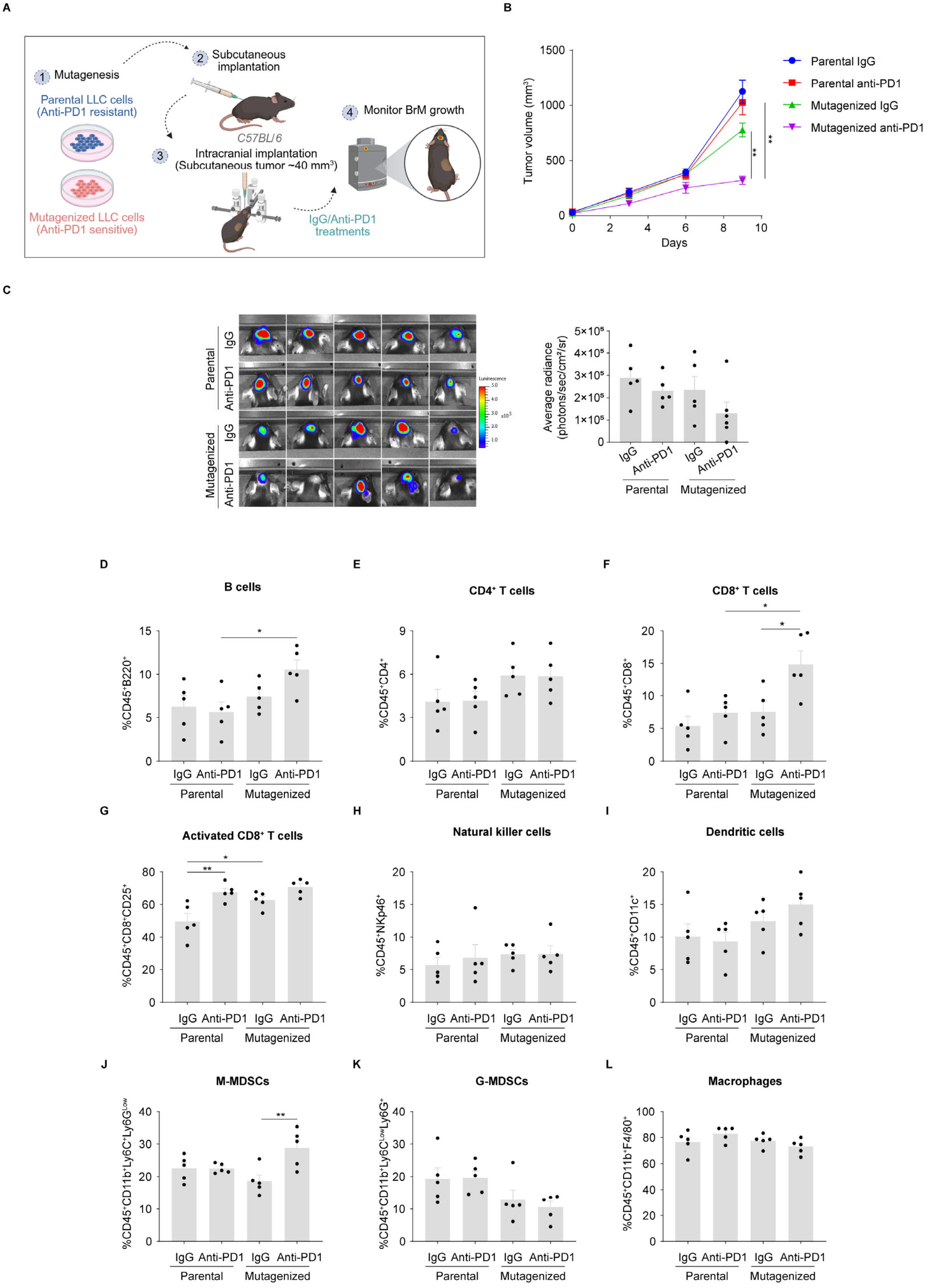
A
1 Mutagenesis
Parental LLC cells
(Anti-PD1 resistant)
Mutagenized LLC cells
(Anti-PD1 sensitive)
2 Subcutaneous implantation
C57BL/6
3 Intracranial implantation
(Subcutaneous tumor ~40 mm³)
IgG/Anti-PD1 treatments
4 Monitor BrM growth
B
Tumor volume (mm³)
Days
Parental IgG
Parental anti-PD1
Mutagenized IgG
Mutagenized anti-PD1
C
Parental
Mutagenized
IgG
Anti-PD1
Luminescence
Average radiance (photons/sec/cm²/sr)
D
B cells
%CD45⁺B220⁺
E
CD4⁺ T cells
%CD45⁺CD4⁺
F
CD8⁺ T cells
%CD45⁺CD8⁺
G
Activated CD8⁺ T cells
%CD45⁺CD8⁺CD25⁺
H
Natural killer cells
%CD45⁺NKp46⁺
I
Dendritic cells
%CD45⁺CD11c⁺
J
M-MDSCs
%CD45⁺CD11b⁺Ly6C⁺Ly6G^Low
K
G-MDSCs
%CD45⁺CD11b⁺Ly6C^Low Ly6G⁺
L
Macrophages
%CD45⁺CD11b⁺F4/80⁺

Fig. S15. Effect of anti-PD1 therapy on the intracranial progression of sensitive vs. resistant lung cancer cells. Eight-week-old C57BL/6 mice bearing subcutaneous xenografts of either anti-PD1-resistant (parental) or anti-PD1-sensitive (mutagenized) LLC cells were used in a dual-site implantation model. Once subcutaneous tumors reached ~40 mm^3, the corresponding cell type was implanted intracranially, and mice were treated with anti-PD1 or IgG control for one week starting on the day of intracranial implantation. **(A)** Schematic of the dual-site implantation model and experimental design **(B-C)** Subcutaneous tumor growth curves (B), and bioluminescence images (C) showing intracranial tumor burden across treatment groups (n=5 mice per group). At the end of treatment, mice were perfused and brains were processed into single-cell suspensions for flow cytometric profiling of brain-infiltrating immune cells. Bar graphs show the percentage of **(D)** B cells, **(E)** $CD4^+$ T cells, **(F)** $CD8^+$ T cells, **(G)** activated $CD8^+$ T cells ($CD8^+CD25^+$), **(H)** natural killer cells, **(I)** dendritic cells, **(J)** monocytic-MDSCs (M-MDSCs), **(K)** granulocytic-MDSCs (G-MDSCs), and **(L)** macrophages in intracranial tumors under different conditions are plotted (n=5 mice/group). Statistical significance was determined by one-way ANOVA (*$p < 0.05$; **$p < 0.01$). (A, created in BioRender. Raviv, Z. (2026) https://BioRender.com/dsome9u).
