# Supplementary material for: PD-1 Blockade–Induced DKK1 Expression by CD8+ T Cells Promotes Blood–Brain Barrier Permeabilization
Source: Cancer Discov. 2026 Jan 13;16(5):976–92. doi: 10.1158/2159-8290.CD-25-1222 (PMC13133603; doi:10.1158/2159-8290.CD-25-1222)
Supplement: Supplementary Figure 16 — Chemotherapy does not alter the permeability of the blood-brain barrier [file cd-25-1222_supplementary_figure_16_suppsf16.pdf]

FIGURE S16

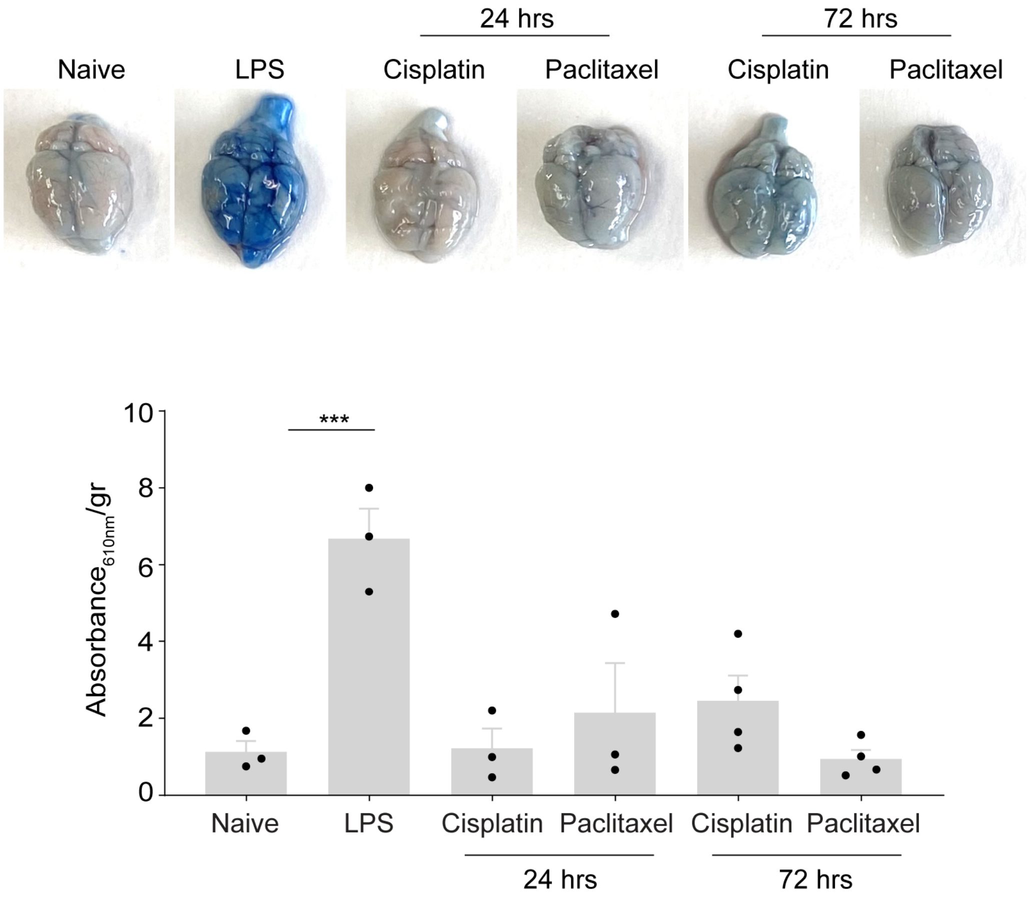

Figure S16: Chemotherapy does not alter the permeability of the blood-brain barrier. Eight-week-old BALB/c mice were administered with a single dose of LPS, cisplatin or paclitaxel, the latter two were administered at the maximum tolerated dose (n=3 mice/group). After 24 (for LPS and both chemotherapy drugs) or 72 hours (for both chemotherapy drugs), the brains were perfused with Evans blue (EB) to evaluate the blood-brain barrier (BBB) permeability. Representative images of perfused brains are shown. Absorbance of EB dye extracted per gram of brain tissue is plotted. Significance was assessed by means of a one-way ANOVA (****p*<0.05).
